# Chiral Nanoparticles Suppress Inflammatory Infiltration to Promote Extracellular Matrix Remodeling for Ectopia Lentis Therapy

**DOI:** 10.1002/advs.202522741

**Published:** 2026-07-27

**Authors:** Yinuo Wen, Yan Yang, Haihan Gao, Yan Liu, Xinyue Wang, Linghao Song, Ruohong Li, Ao Miao, Jie Xu, Zhennan Zhao, Dan Li, Shenjie Peng, Linzhao Li, Min Zhang, Yonghang Liu, Liren Wang, Tianhui Chen, Yongxiang Jiang

**Affiliations:** ^1^ Eye Institute and Department of Ophthalmology Eye & ENT Hospital Fudan University Shanghai China; ^2^ Key Laboratory of Myopia and Related Eye Diseases, NHC; Key Laboratory of Myopia and Related Eye Diseases Chinese Academy of Medical Sciences Shanghai China; ^3^ Shanghai Key Laboratory of Visual Impairment and Restoration Shanghai China; ^4^ Shanghai Medical College (SHMC) Fudan University Shanghai China; ^5^ Guangdong‐Hong Kong Joint Laboratory for Water Security Beijing Normal University Zhuhai China; ^6^ Center For Water Research Advanced Institute of Natural Sciences Beijing Normal University Zhuhai China; ^7^ Department of Sports Medicine Department of Orthopedic Surgery Shanghai Institute of Microsurgery on Extremities Shanghai Sixth People's Hospital Affiliated to Shanghai Jiao Tong University School of Medicine Shanghai China; ^8^ School of Pharmacy and State Key Laboratory of Quality Research in Chinese Medicine Macau University of Science and Technology Macao China; ^9^ National Center for Translational Medicine (Shanghai) SHU Branch Shanghai University Shanghai China

**Keywords:** chiral nanoparticles, ectopia lentis, extracellular matrix remodeling, immunoregulation, zonular fiber reconstruction

## Abstract

Chirality, a fundamental geometric property of matter, plays a pivotal role in biological systems by mediating stereoselective molecular interactions and cellular regulation. Inspired by this principle, chiral nanoparticles have emerged as promising candidates for immunomodulation in inflammation‐related diseases. However, whether and how their chiral cues regulate extracellular matrix (ECM) remodeling remains largely unexplored. Herein, we report the design of L‐cysteine‐configured polyurethane nanoparticles (L‐CN) through covalent grafting of naturally chiral L‐cysteine, and demonstrate their therapeutic potential for ectopia lentis (EL) — a disease characterized by progressive zonular fiber degeneration. Mechanistically, L‐CN suppresses the nuclear factor kappa B signaling pathway in M_1_ macrophages through the anti‐inflammatory activity of L‐cysteine, thereby attenuating inflammatory responses and reducing ECM degradation in vitro. In an EL rat model, it facilitates CD163^+^ M_2_ macrophages polarization, leading to the restoration of FBN1 and MFAP2 expression in zonular fibers and ultimately promoting zonular fiber reconstruction. Overall, this study establishes a chirality‐mediated immunomodulatory strategy for ECM reconstruction and provides a minimally invasive therapeutic approach for EL and potentially other matrix‐degenerative disorders.

## Graphical abstract

L‐cysteine‐configured chiral polyurethane nanoparticles suppress ocular inflammation and reduce extracellular matrix (ECM) degradation in ectopia lentis by regulating macrophages polarization and inhibiting nuclear factor kappa B signaling pathway. By promoting zonular fiber‐associated protein restoration and tissue repair, this minimally invasive strategy offers a promising therapeutic approach for inflammatory and degenerative diseases involving ECM dysfunction.

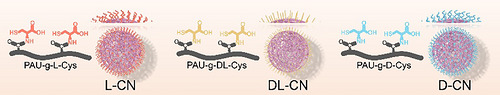

## 1 Introduction

Chirality, defined as the geometric property of a structure being non‐superimposable on its mirror image, is closely related to molecular recognition and functional regulation in biological systems. Precise chiral recognition between biomolecules enables highly specific interactions and coordinated biological processes, thereby underpinning the orderly progression of life activities [1, 2, 3]. In recent years, the rapid development of nanomaterials for biomedical applications has stimulated growing interest in chiral nanoparticles, which can interact with the biological environment, including cell surface receptors and the extracellular matrix (ECM) in a stereoselective manner [4, 5]. Notably, chiral nanomaterials have demonstrated unique advantages in the treatment of inflammatory diseases because of their capacity to mediate stereospecific interactions and regulate immune responses [6, 7, 8, 9]. In particular, they can modulate macrophages polarization between pro‐inflammatory (M_1_) and anti‐inflammatory (M_2_) phenotypes, thereby providing new opportunities for targeted intervention in inflammation‐related disorders [10, 11, 12].

Ectopia lentis (EL), a progressive and vision‐threatening ocular disorder, represents a disease in urgent need of such mechanism‐based therapeutic strategies. Approximately 60% of EL cases occur during early childhood (ages 3–5), and the disease is characterized by gradual degeneration or rupture of zonular fibers, ultimately leading to severe and irreversible visual impairment [13, 14, 15, 16]. Increasing evidence suggests that the pathogenesis of EL involves progressive ECM degradation, particularly accompanied by significant downregulation of fibrillin‐1 (FBN1) expression [17, 18, 19]. Currently, surgical reconstruction of the zonular fibers remains the primary clinical treatment option. However, this approach is associated with considerable trauma, high surgical complexity, and substantial risk of complications (such as glaucoma and retinal detachment), which makes this approach insufficient for addressing long‐term disease management [20, 21]. Therefore, the development of minimally invasive therapeutic strategies that directly target the underlying pathological mechanisms remains highly desirable.

Recent studies have further identified chronic immune‐mediated inflammation as a key driver of pathological zonular degeneration in EL [13, 22]. Under physiological conditions, the structural integrity of the zonular fibers depends on an ECM network enriched with FBN1 and microfibril‐associated protein 2 (MFAP2) [23, 24]. Approximately 85% of EL patients exhibit significantly reduced FBN1 expression, which is closely related to persistent inflammatory activation and progressive ECM degradation. Chronic inflammation promotes the upregulation of matrix metalloproteinases (MMPs), including MMP‐2 and MMP‐9, and increases oxidative stress, collectively accelerating ECM breakdown and ultimately causing irreversible structural damage to the zonular fibers [13, 22, 25, 26]. Consequently, precise regulation of the ocular inflammatory microenvironment and restoration of ECM homeostasis have emerged as central challenges for achieving effective zonular repair. With this pathological context, chiral nanoparticles offer a rational strategy for EL treatment. Through stereoselective interactions with biomolecules, these nanoparticles may precisely regulate cellular signaling pathways and immune responses, thereby suppressing chronic inflammation and reducing ECM degradation. In addition, their excellent biocompatibility and potential for localized delivery make them attractive candidates for minimally invasive ocular therapy [27]. Among various chiral biomolecules, cysteine (Cys), a naturally occurring sulfur‐containing amino acid, has attracted particular interest because of its anti‐inflammatory, antioxidant, and biocompatible properties. Previous studies have demonstrated that Cys‐based chiral materials can regulate immune responses and tissue repair. For example, incorporation of Cys enantiomers into chiral flower‐like aluminum oxyhydroxide supraparticles was shown to enhance humoral immune response [28]. However, no studies have reported the use of chiral Cys‐based nanoparticles for ocular disease therapy through localized ocular administration.

**SCHEME 1:**
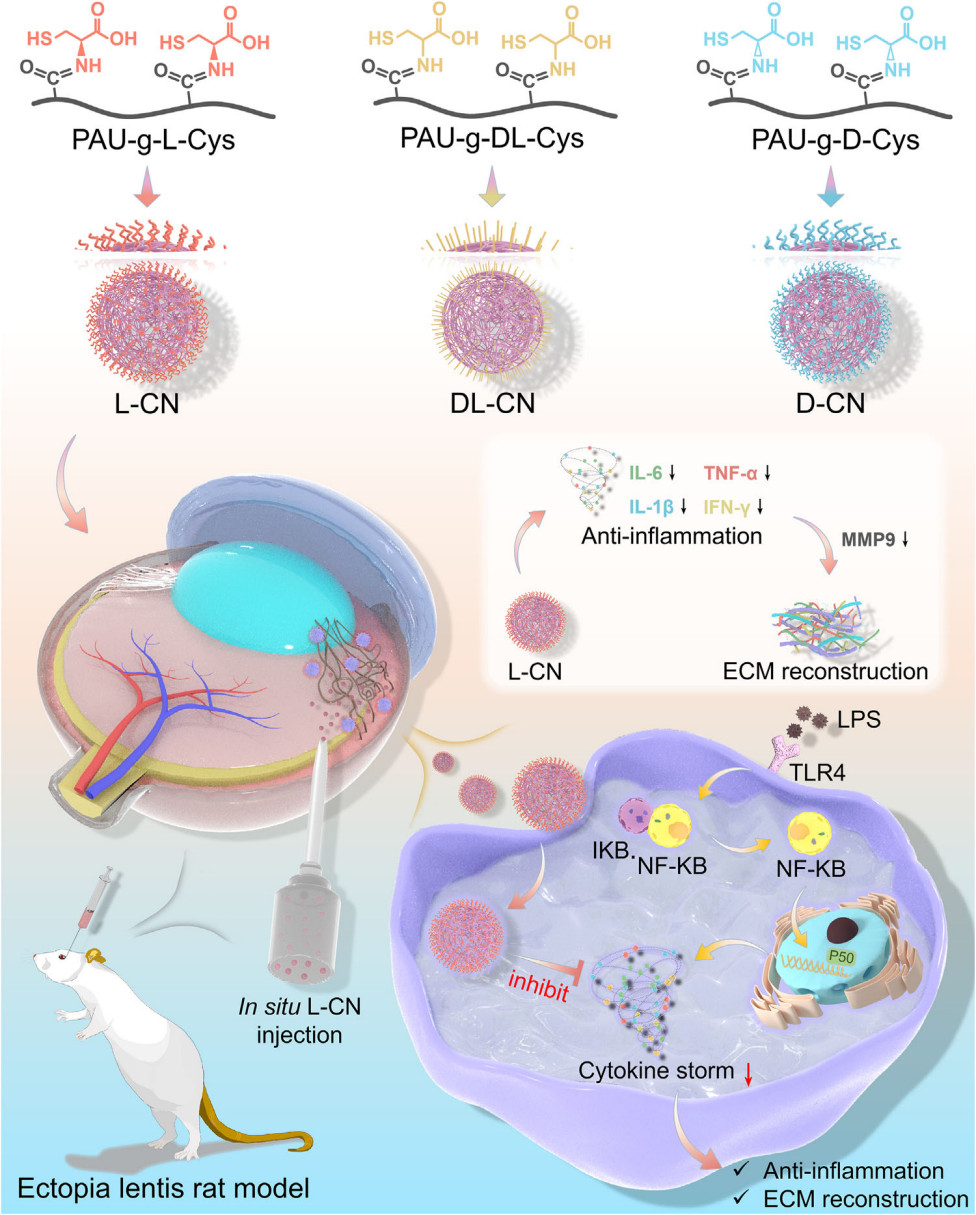
L‐CN injection to promote the repair of zonular fiber structures in an EL rat model.

Polyurethane (PAU/PU), a block copolymer composed of alternating soft‐hard segment structure, has emerged as a promising biomaterial due to its excellent designability, biodegradability, and biosafety [29, 30]. More critically, its microphase‐separated architecture partially recapitulates the intertwined organization of collagen fibers and proteoglycans in the native ECM, making it highly attractive for tissue repair and regenerative applications [31, 32]. Based on these considerations, we grafted naturally chiral L‐cysteine (L‐Cys) onto a biodegradable PAU backbone through graft copolymerization to construct L‐cysteine‐configured polyurethane nanoparticles (L‐CN) for EL treatment. Compared with conventional anti‐inflammatory eye drops, which typically exhibit limited corneal penetration efficiency (≈ 5–10%), intravitreally administered anti‐inflammatory nanomaterials enable improved posterior segment delivery and prolonged intraocular retention [33]. Our results demonstrated that L‐CN, in comparison to D‐cysteine‐configured polyurethane nanoparticles (D‐CN) and DL‐cysteine‐configured polyurethane nanoparticles (DL‐CN), exhibited superior biological activity. Specifically, L‐CN significantly promoted the proliferation and migration of human lens epithelial cells (HLECs) in vitro and concurrently suppressed the expression of inflammatory cytokines (e.g. IL‐6, IL‐1β, and TNF‐α) by inhibiting nuclear factor kappa B (NF‐κB) nuclear translocation, thereby contributing to ECM reconstruction (Scheme 1). Moreover, single‐cell RNA sequencing (scRNA‐seq) analysis in an EL rat repair model revealed that L‐CN treatment increased the abundance of anti‐inflammatory cells and promoted functional recovery of non‐pigmented ciliary epithelial cells (NPCECs) and lens epithelial cells (LECs. Collectively, this work establishes a chiral nanoparticle‐based strategy for precise immunomodulation and ECM repair, providing a minimally invasive therapeutic strategy for EL, and highlighting the broader potential of chiral biomaterials in tissue repair and disease treatment.

## 2 Results

### 2.1 Synthesis, Characterization, and Cytotoxicity Assessment of Chiral Nanoparticles

**FIGURE 1:**
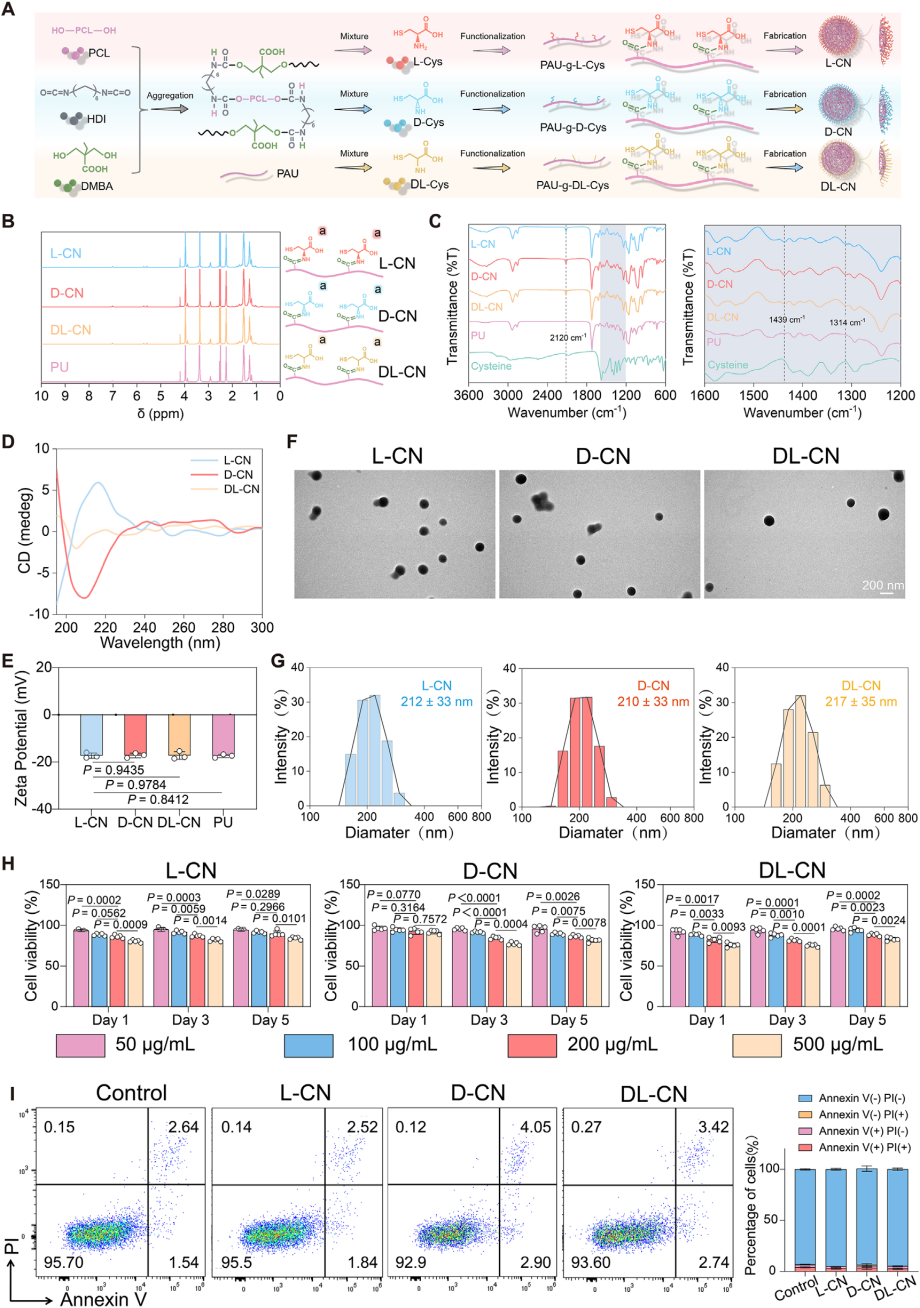
Synthesis and biocompatibility evaluation of L‐CN, D‐CN, and DL‐CN. (A) Schematic illustration of the preparation of PU functionally modified with L‐Cys, D‐Cys, and DL‐Cys. (B) ^1^H NMR spectra of L‐CN (blue curve), D‐CN (red curve), DL‐CN (yellow curve), and PU (pink curve). (C) FTIR spectra of L‐CN, D‐CN, DL‐CN, PU, and Cysteine (green curve). (D) CD analysis of L‐CN, D‐CN, and DL‐CN. (E) Zeta potential L‐CN, D‐CN, DL‐CN, and PU. (F) Microscopic structure of L‐CN, D‐CN, and DL‐CN. Scale bar, 200 nm. (G) DLS analysis of L‐CN, D‐CN, and DL‐CN. (H) Cell viability in different groups in CCK‐8 assays from day 1 to day 5 (*n* = 5). (I) Flow cytometry scatter plot images and statistical results of HLECs treated with L‐CN, D‐CN, and DL‐CN in annexin V/PI test (*n* = 3). Statistical analyses were performed using One‐way Analysis of Variance (ANOVA) followed by Tukey's multiple comparisons tests. Data are presented as mean ± SD.

We synthesized functionalized PAU nanoparticles through a two‐step polymerization strategy. The pre‐polymerization reaction was performed using polycaprolactone diol (PCL‐OH) and hexamethylene diisocyanate (HDI), followed by chain extension with dimethylolbutanoic acid (DMBA) to generate the base polymer. Subsequent covalent conjugation with L‐Cys, D‐cysteine (D‐Cys), or DL‐cysteine (DL‐Cys) yielded the corresponding chiral nanoparticles, termed L‐CN, D‐CN, and DL‐CN, respectively (Figure 1A). As shown in Figure 1B, the chiral nanoparticles were characterized by ^1^H nuclear magnetic resonance (^1^H‐NMR) spectroscopy. Following functionalization, a characteristic Cys‐associated peak appeared at 5.5 ppm, confirming successful modification. Further characterization by fourier transform infrared spectroscopy (FTIR) showed that the characteristic S‐H stretching vibration peak of Cys, typically observed at 2550–2600 cm^−^^1^, disappeared after grafting, indicating the consumption of thiol groups during the amidation process. A new absorption peak corresponding to the combination frequency of amide bonds appeared at 2120 cm^−^^1^. Collectively, these spectral changes confirmed the successful grafting of Cys onto the PAU backbone through amide linkage formation (Figure 1C). Circular dichroism (CD) spectroscopy further verified the chiral configurations, with L‐CN exhibiting a positive Cotton effect at 216 nm and D‐CN showing a mirror‐image negative Cotton effect, indicating opposite surface chiral configurations. In contrast, DL‐CN showed no obvious characteristic peak because of its racemic nature (Figure 1D). As demonstrated in Figure 1E, the zeta potentials of L‐CN, D‐CN, and DL‐CN were −17.3 ± 1.2, −17.2 ± 1.0, and −17.3 ± 1.6 mV, respectively, with no significant difference compared to the non‐chiral PAU nanoparticles (−17.3 ± 1.6 mV, *p* > 0.05). These results suggested that the Cys modification did not substantially alter the surface physicochemical properties of the PAU nanoparticles, thereby minimizing potential interference in subsequent studies of chiral nanoparticle‐cell interactions. Transmission electron microscopy (TEM) images showed that all three nanoparticles possessed uniform, smooth, and spherical morphologies (Figure 1F), with dynamic light scattering (DLS) analysis illustrating comparable hydrated diameters (L‐CN: 212 ± 33 nm, D‐CN: 210 ± 33 nm, DL‐CN: 217 ± 35 nm, respectively) (Figure 1G). We further evaluated the stability of L‐CN under different storage conditions, and the nanoparticle stability was monitored at both 25°C and 37°C (Figure S1). Considering the pH fluctuations associated with ocular inflammation (pH 7.1–7.5) [34], the particle size was further evaluated under physiologically relevant conditions at pH 7.1 and pH 7.5. Only negligible variations in particle size were observed, indicating excellent colloidal stability across the inflammatory ocular microenvironment and supporting its suitability for ocular applications.

Biological evaluations demonstrated excellent biocompatibility of the nanoparticles. At a concentration of 200 µg/mL, none of the nanoparticles showed significant cytotoxicity toward HLECs, human corneal epithelial cells (HCECs), and human trabecular meshwork cells (HTMCs) (Figure 1H and Figures S2A–D and S3A). Live/dead staining and flow cytometry further confirmed minimal cell death following 5 days of exposure (Figure 1I and Figure S3A). Notably, L‐CN significantly promoted HLECs migration in both horizontal (Figure S3B–D) and vertical (Figure S3E,F) migration assays compared with D‐CN and DL‐CN, suggesting chirality‐dependent biological activity. Collectively, these results showed the favorable biocompatibility and therapeutic potential of the chiral nanoparticles.

### 2.2 Multi‐omics Reveal Inflammation and ECM Dysregulation in EL Pathogenesis

**FIGURE 2:**
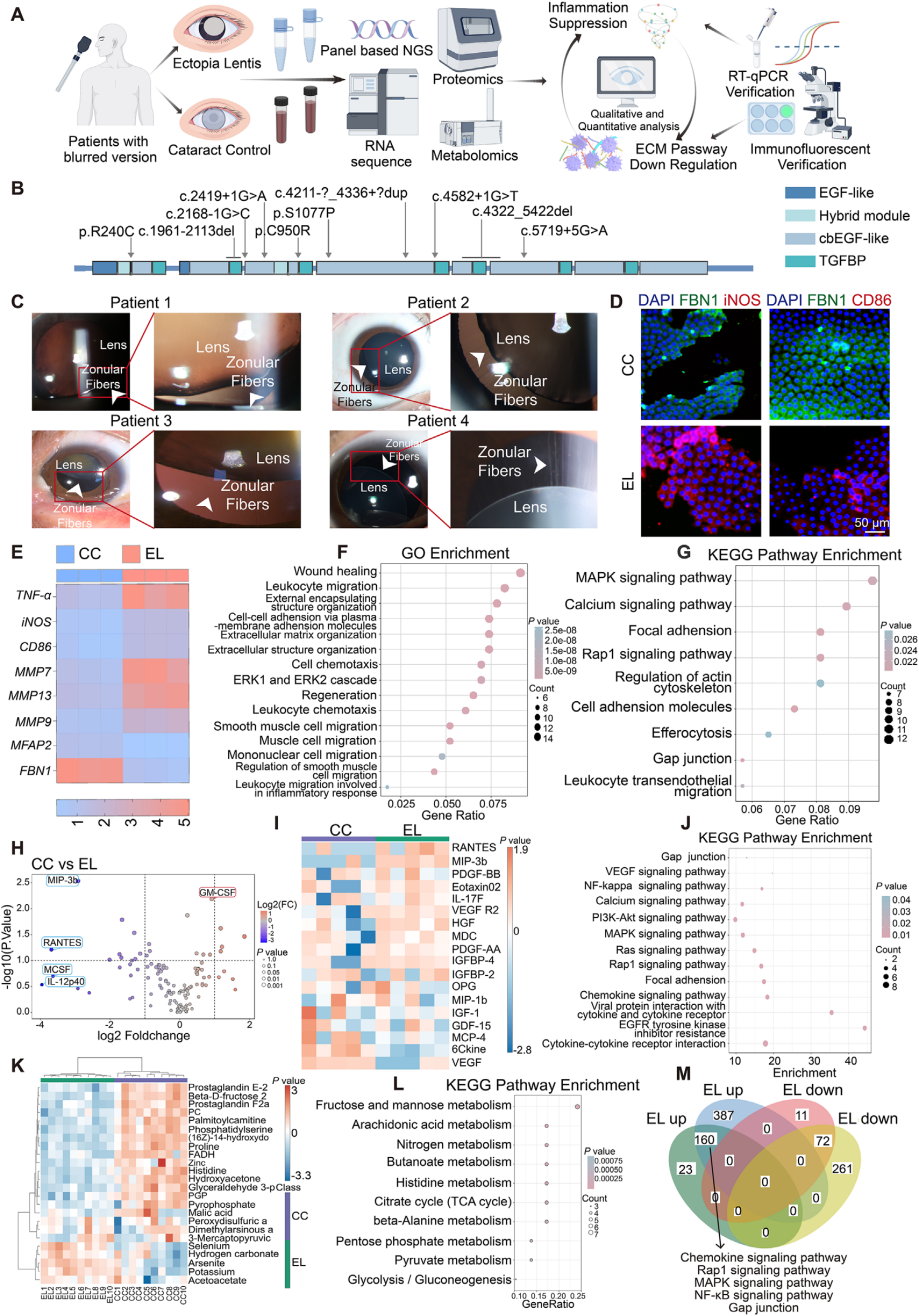
Evaluation of multi‐omics analysis in patients with EL. (A) Schematic representation of the clinical sample testing workflow for EL and CC patients. (B) The panel‐based NGS analysis of peripheral blood samples revealed common mutation sites in the *FBN1* gene. (C) Representative slit‐lamp anterior segment photographs of EL patients across severity grades. (D) IF staining for FBN1, iNOS, and CD86 of capsular tissues from EL and CC patient tissues (*n* = 3). Scale bar, 50 µm. (E) Comparative RT‐qPCR analysis of relative genes expression in EL and CC patient tissues (*n* = 3). (F) GO enrichment analysis results for differentially expressed genes between CC and EL patient samples. (G) KEGG enrichment analysis of pathways enriched for genes differentially expressed in CC and EL patients. (H) Proteomic profiling of aqueous humor revealed significant abundance variations between CC and EL patient cohorts. (I) Protein clustering analysis of CC and EL patients. (J) KEGG enrichment analysis of differentially abundant proteins identified in CC and EL patients. (K) Heatmap of key differentially expressed metabolites in CC versus EL samples. (L) KEGG analysis of significant differentially expressed metabolites. (M) Venn diagram of multi‐omics integrated analysis (Blue indicated pathways upregulated in EL in RNA‐seq, green indicated pathways upregulated in EL in proteomics, yellow indicated pathways downregulated in EL in RNA‐seq, and red indicated pathways downregulated in EL in proteomics). Statistical analyses were performed using One‐way ANOVA followed by Tukey's multiple comparisons tests and unpaired two‐tailed Student's t test. Data are presented as mean ± SD.

A cohort of 100 patients diagnosed with EL from the Department of Ophthalmology, Eye and ENT Hospital of Fudan University, was enrolled in this study (Ethics No. ChiCTR2000039132). Genetic analysis identified *FBN1* mutations in 92.00% of cases (92/100). Panel‐based next‐generation sequencing (NGS) further revealed 10 mutation sites clustered within four structural domains of *FBN1* (Figure 2A,B), suggesting that structural abnormalities of the FBN1 protein contributed to EL pathogenesis. Clinical examination of representative anterior segment photographs from EL patients showed varying degrees and quadrants of lens dislocation (Figure 2C). To further investigate the pathological alterations associated with EL, anterior capsule tissues from both EL patients and control age‐related cataract (CC) patients were analyzed by reverse transcription quantitative polymerase chain reaction (RT‐qPCR) (Figure 2E) and immunofluorescence (IF) staining (Figure 2D and Figure S4A–C). The results revealed significant downregulation of *FBN1* and *MFAP2* expression in EL tissues, accompanied by increased expression of inflammatory markers, including *TNF‐α, iNOS*, and *CD86*, as well as *MMP7, MMP9, and MMP13*. To further validate these findings at the transcriptomic level, bulk RNA sequencing (RNA‐seq) was performed using anterior capsule tissues from EL and CC patients (Figure S5A). Mullany et al. previously employed RNA‐seq analysis on anterior lens capsule epithelial tissues from pseudoexfoliation syndrome and CC patients, which supported the suitability of cataract samples as controls for transcriptomic analysis [35]. Differential expression analysis identified 559 upregulated genes in EL tissues, including inflammatory mediators (*LCN*, *LCP1*), chemokines (*CX3CL1*), and matrix metalloproteinases (*MMP28*), together with 321 downregulated genes associated with ECM organization and remodeling, such as *COL11A2*, *FLRT2* (Figure S5B,C). Gene Ontology (GO) and Kyoto Encyclopedia of Genes and Genomes (KEGG) enrichment analyses demonstrated significant enrichment of these differentially expressed genes (DEGs) in biological processes related to ECM remodeling, intercellular communication, and immune cell trafficking (Figure 2F,G and Figure S5D), supporting the coexistence of ECM degradation and chronic inflammation in EL pathology (Figure S5E). Proteomic analysis of aqueous humor further identified 120 differentially expressed proteins, predominantly associated with ECM remodeling and inflammatory responses (Figure 2H,I and Figure S5F,G). These proteins were significantly enriched in pathways related to immune cell migration, chemotaxis, and cytokine‐receptor interactions (Figure 2J and Figure S5H). In parallel, non‐targeted metabolomic analysis of aqueous humor revealed elevated levels of arachidonic acid metabolites and nitrogen‐containing compounds in EL patients, further implicating macrophage‐associated inflammatory responses in disease progression [36] (Figure 2K,L and Figure S5I–K). Collectively, these integrated multi‐omics analyses identified chronic inflammation and ECM degradation as key pathological hallmarks of EL (Figure 2M). These results highlighted the therapeutic potential of simultaneously suppressing inflammation and promoting ECM regeneration, thereby providing a rational strategy for EL treatment.

### 2.3 Phagocytic Cell Uptake of Chiral Nanoparticles

**FIGURE 3:**
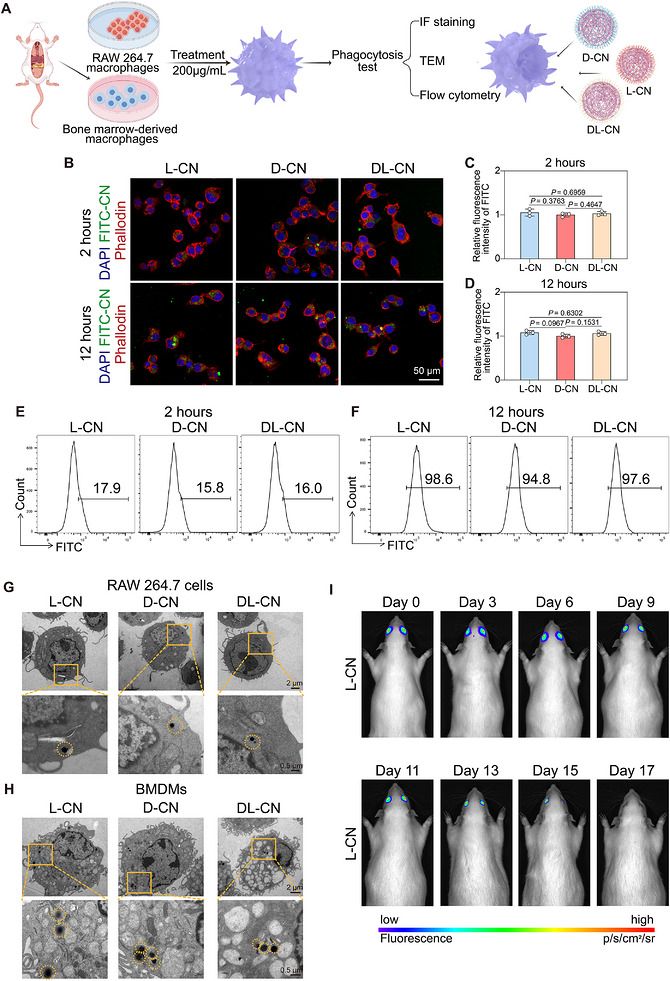
In vitro and in vivo metabolic process of L‐CN, D‐CN, and DL‐CN. (A) Schematic diagram of the cellular uptake. B‐D) IF staining of RAW 264.7 cells after being co‐cultured with FITC‐labeled L‐CN, D‐CN, and DL‐CN for 2 and 12 h (*n* = 3). Scale bar, 50 µm. (E,F) Flow cytometry of FITC‐labeled L‐CN, D‐CN, and DL‐CN after being co‐cultured with RAW 264.7 cells for 2 and 12 h. (G,H) Representative images of TEM in vitro cell uptake of L‐CN, D‐CN, and DL‐CN (yellow dashed circles) in RAW 264.7 cells and BMDMs after 2 h (*n* = 3). Scale bar, 2 µm, and 0.5 µm. (I) In vivo real‐time fluorescence imaging of rats treated with L‐CN over a 17‐day observation period (*n* = 3). Statistical analyses were performed using One‐way ANOVA followed by Tukey's multiple comparisons test. Data are presented as mean ± SD.

Fluorescein isothiocyanate (FITC)‐labeled nanoparticles (FITC‐L‐CN, FITC‐D‐CN, and FITC‐DL‐CN) were used to evaluate nanoparticle uptake by RAW 264.7 cells (Figure 3A). IF imaging after 2 h of incubation showed comparable cell‐associated fluorescence signals among the three nanoparticle groups (Figure 3B,C). Upon extending the incubation time to 12 h, markedly increased fluorescence signals associated with cells were observed in all groups (Figure 3B,D), indicating enhanced nanoparticle‐cell interactions over time. Quantitative flow cytometry analysis further revealed a clear time‐dependent increase in nanoparticle‐associated fluorescence. Specifically, the percentage of fluorescence‐positive RAW 264.7 cells exposed to FITC‐L‐CN increased from 17.90% at 2 h to 98.60% at 12 h. Similar uptake behaviors were also observed for the D‐CN and DL‐CN groups (Figure 3E,F). TEM further provided direct ultrastructural evidence of nanoparticles internalization in both RAW 264.7 cells and primary bone marrow‐derived macrophages (BMDMs) following 2 h of co‐culture, confirming that the nanoparticles were successfully internalized rather than merely attached to the cell surface (Figure 3G,H). In vivo imaging was performed to evaluate the retention of different chiral nanoparticles after a single intravitreal injection. All Cy7‐labeled nanoparticles showed clear ocular fluorescence signals, which remained detectable up to day 13. By day 17, fluorescence signals had nearly disappeared in all groups, indicating effective intraocular clearance without obvious long‐term accumulation (Figure 3I and Figure S6A,B).

### 2.4 Evaluation of L‐CN on Polarization of Macrophages

**FIGURE 4:**
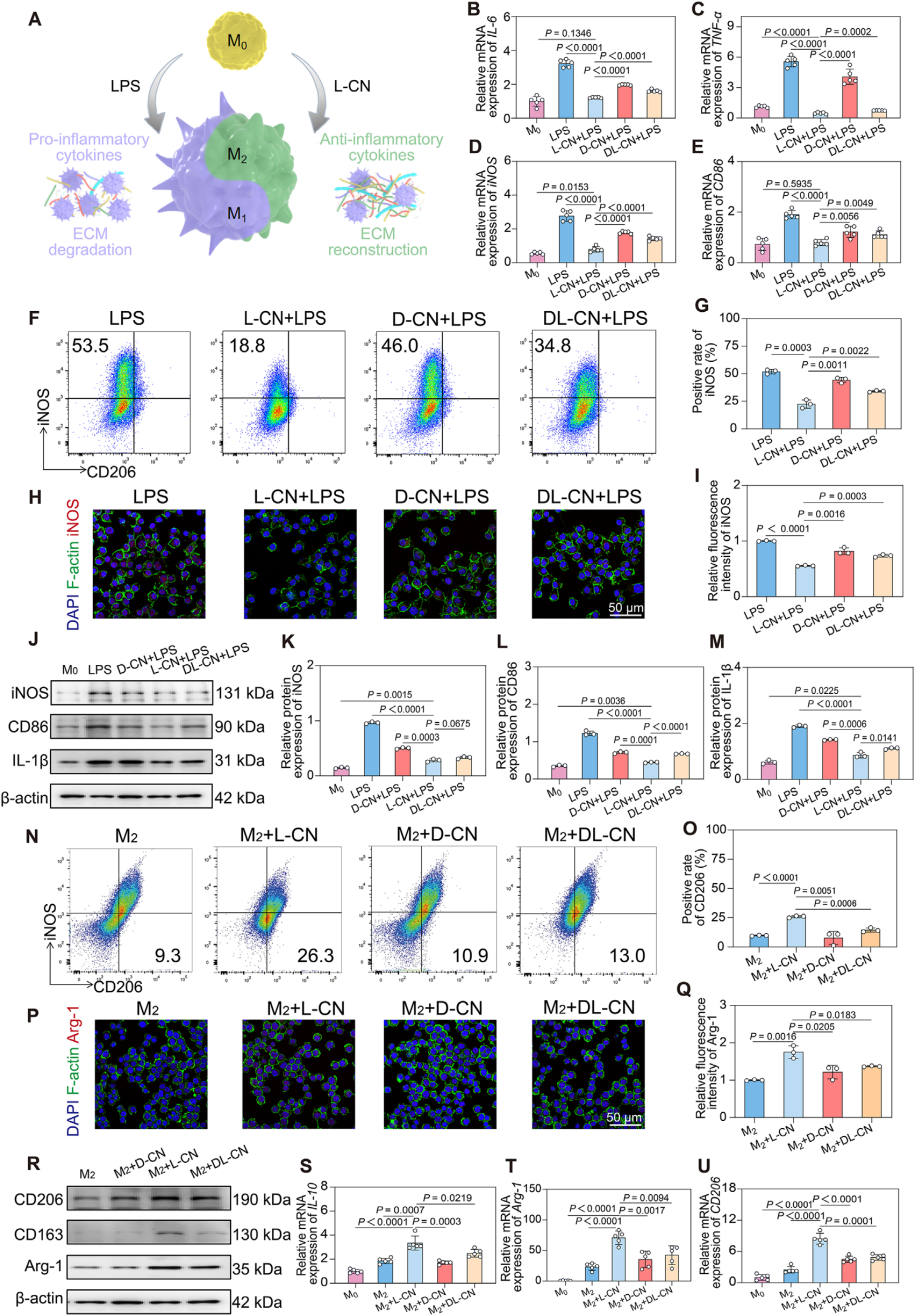
Immunomodulatory effects of L‐CN, D‐CN, and DL‐CN. (A) Schematic illustration of L‐CN‐mediated macrophages polarization. (B–E) RT‐qPCR‐based analysis of the effects of L‐CN, D‐CN, and DL‐CN on macrophages polarization under M_1_ induction condition (*n* = 5). (F,G) Flow cytometry of iNOS in RAW 264.7 cells under M_1_ induction condition (*n* = 3). (H,I) IF staining for iNOS in RAW 264.7 cells under M_1_ induction condition and the relative fluorescence intensity (*n* = 3). Scale bar, 50 µm. (J–M) Western blot analysis showed the expression of M_1_‐associated markers in different groups and the quantitative analysis showed the results (*n* = 3). (N,O) Flow cytometry of CD206 in RAW 264.7 cells under M_2_ induction condition (*n* = 3). (P,Q) IF staining for Arg‐1 in RAW 264.7 cells under M_2_ induction condition and the relative fluorescence intensity (*n* = 3). Scale bar, 50 µm. (R) Western blot analysis showed the expression of M_2_‐associated markers in different groups (*n* = 3). (S–U) Gene expression of RAW 264.7 cells under M_2_ induction condition (*n* = 5). Statistical analyses were performed using One‐way ANOVA followed by Tukey's multiple comparisons test. Data are presented as mean ± SD.

We next investigated the effects of the three chiral nanoparticles on macrophages polarization (Figure 4A). RT‐qPCR demonstrated that L‐CN significantly suppressed the expression of M_1_ macrophages‐associated genes (*IL‐6*, *TNF‐α*, *iNOS*, *CD86*, and *IL‐1β*) (Figure 4B–E and Figure S7). Flow cytometry further showed that L‐CN treatment reduced the proportion of iNOS^+^ M_1_ macrophages from 51.90 ± 2.04% to 22.50 ± 3.80% (Figure 4F,G). Consistently, IF staining confirmed a marked decrease in iNOS^+^ macrophages following L‐CN treatment (L‐CN: 55.37 ± 0.87%, D‐CN: 81.68 ± 5.96%, DL‐CN: 73.35 ± 2.43%, respectively; Figure 4H,I). Western blot (WB) analysis further revealed that L‐CN significantly downregulated the expression of M_1_‐related proteins, including iNOS, CD86, and IL‐1β (*p* < 0.05; Figure 4J–M). Then, we attempted to examine whether L‐CN could promote M_2_ macrophages polarization. Upon addition of L‐CN to the M_2_ induction medium, the proportion of CD206^+^ (M_2_) macrophages increased significantly from 9.71 ± 1.49% to 26.80 ± 1.73% (Figure 4N,O). IF analysis also showed that L‐CN markedly increased the expression of Arg‐1^+^ macrophages (Figure 4P,Q). Additionally, M_2_‐associated markers (CD206, CD163, and Arg‐1) were significantly upregulated following L‐CN treatment (Figure 4R and Figure S8A–C) (*p* < 0.05). Moreover, enzyme‐linked immunosorbent assay (ELISA) analysis revealed that L‐CN treatment induced significantly higher secretion of the anti‐inflammatory cytokines TGF‐β, IL‐10, and IL‐13 than D‐CN and DL‐CN treatment, consistent with a more pronounced M_2_‐polarized macrophages phenotype (Figure S9A–C). These findings were further corroborated by RT‐qPCR analysis (Figure 4S–U). Notably, L‐CN exhibited superior immunomodulatory effects compared to both D‐CN and DL‐CN, indicating chirality‐associated differences in the regulation of macrophages polarization.

### 2.5 L‐CN Protects the Zonular Fiber‐Related Genes Expression of HLECs

**FIGURE 5:**
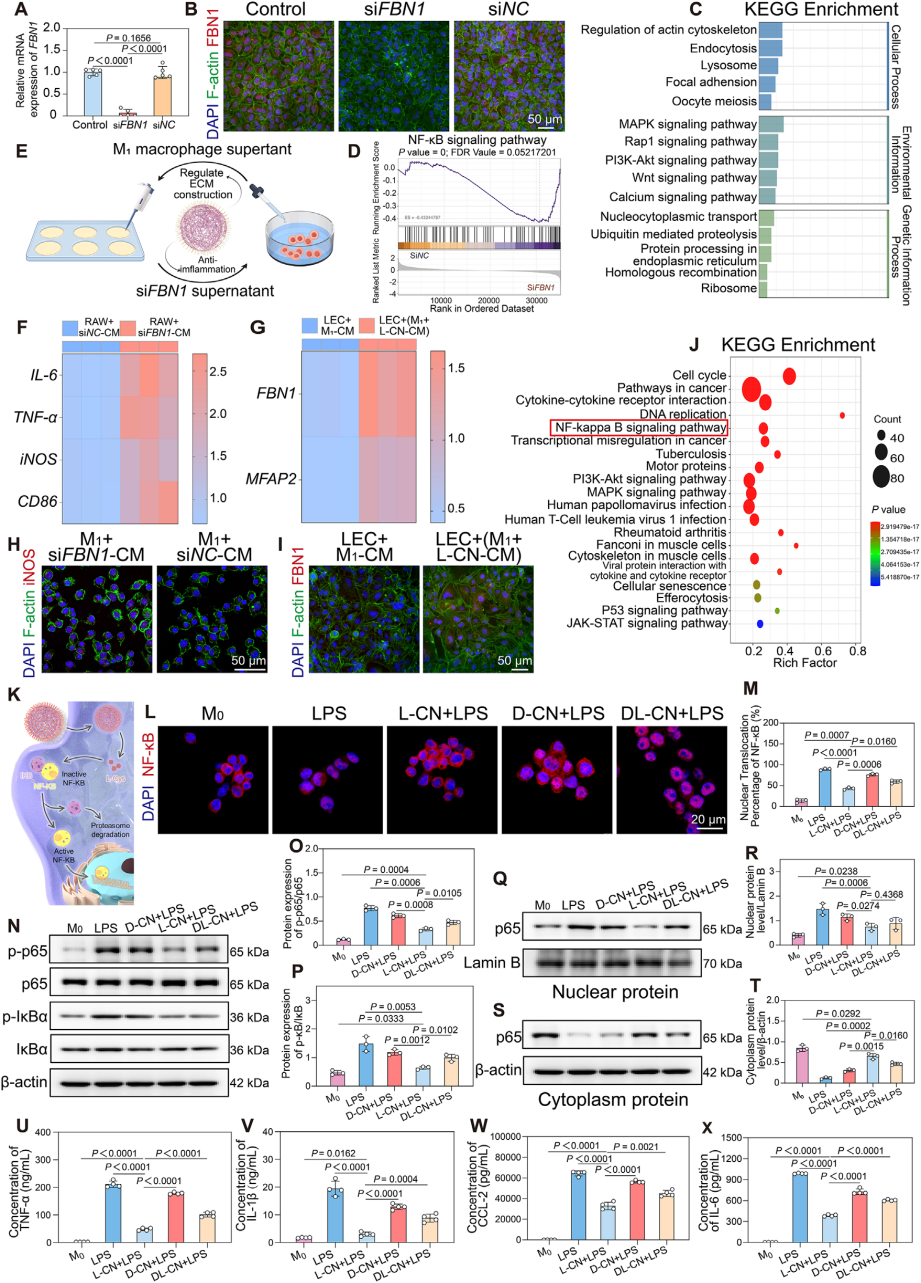
ECM reconstruction evaluation of L‐CN. (A) RT‐qPCR showed the gene expression of HLECs after transfection with *FBN1*‐targeting siRNA (*n* = 5). (B) IF staining of FBN1 in HLECs after transfection with *FBN1*‐targeting siRNA (*n* = 3). Scale bar, 50 µm. (C) KEGG enrichment analysis. (D) Gene set enrichment analysis. (E) Schematic diagram of L‐CN modulated macrophages polarization for providing an ECM‐reconstructed niche. (F,G) RT‐qPCR‐based analysis of the effects of L‐CN on macrophages polarization and ECM reconstruction (*n* = 3). (H) IF staining analysis of iNOS in RAW 264.7 cells in different groups (*n* = 3). Scale bar, 50 µm. (I) IF staining analysis of FBN1 in HLECs in different groups (*n* = 3). Scale bar, 50 µm. (J) KEGG enrichment analysis. (K) Schematic diagram of NF‐κB signaling pathway. (L,M) Quantification of nuclear localization of NF‐κB p65 in different groups (*n* = 3). Scale bar, 20 µm. (N–T) Western blot analysis revealed the expression of transducing proteins (p‐p65, p‐IκBα) and the content of p65 in the cytoplasm and nucleus in different groups (*n* = 3). (U–X) Concentrations of TNF‐α, IL‐1β, CCL‐2, and IL‐6 in the supernatant from each group (*n* = 5). Statistical analyses were performed using One‐way ANOVA followed by Tukey's multiple comparisons test. Data are presented as mean ± SD.

Clinical observations suggested that persistent inflammation continuously affected the zonular fibers, leading to gradual ECM degradation and disease progression. Based on these findings, we further investigated the impact of inflammation on zonular ECM remodeling via an in vitro model. Given the superior anti‐inflammatory efficacy of L‐CN demonstrated in prior experiments, all subsequent in vitro studies were performed with this nanoparticle. To establish an in vitro EL model, HLECs were transfected with *FBN1*‐targeting siRNA, and successful knockdown was confirmed at both transcriptional and protein levels after 48 h of transfection (Figure 5A,B and Figure S10A–C). RNA‐seq analysis revealed significant alterations in MAPK, Rap1, PI3K‐AKT, and Wnt signaling pathways (Figure 5C and Figure S10D–F). Gene set enrichment analysis further supported change of the NF‐κB signaling pathway in the si*FBN1* group (NES = ‐0.43, FDR = 0.05, Figure 5D and Figure S10G), suggesting that ECM degradation might trigger an inflammatory response within the ocular microenvironment. Conditioned medium collected from *FBN1*‐silenced HLECs (si*FBN1*‐CM) markedly increased the expression of M_1_ macrophages‐associated markers (*IL‐6*, *TNF‐α*, *iNOS*, and *CD86*) (Figure 5F). In contrast, L‐CN treatment effectively preserved ECM integrity by maintaining *FBN1* and *MFAP2* expression (Figure 5G). Similar findings were further confirmed by IF staining (Figure 5H,I and Figure S10H,I). Mechanistic investigation through RNA‐seq identified 2048 upregulated genes and 1488 downregulated genes following L‐CN treatment (Figure S11A–C). Pathway enrichment analysis highlighted cytokine‐cytokine receptor interaction, NF‐κB signaling, PI3K‐Akt signaling, and MAPK signaling pathways as major pathways associated with genes altered by L‐CN (Figure 5J and Figure S11D,E). IF staining demonstrated that lipopolysaccharide (LPS) stimulation significantly promoted NF‐κB p65 nuclear translocation, whereas L‐CN treatment effectively inhibited this process (Figure 5K–M). Western blot analysis also demonstrated that L‐CN significantly suppressed LPS‐induced phosphorylation of p65 and IκB, thereby inhibiting activation of the NF‐κB signaling pathway (Figure 5N–P). Inhibition of p65 phosphorylation further prevented its nuclear translocation, resulting in the predominant retention of p65 within the cytoplasm with minimal nuclear localization (Figure 5Q–T). Additionally, the secretion of pro‐inflammatory cytokines (TNF‐α, IL‐1β, CCL‐2, and IL‐6) was significantly attenuated upon L‐CN treatment (Figure 5U–X). These findings established L‐CN suppressed LPS‐induced NF‐κB activation by suppressing NF‐κB p65 phosphorylation and nuclear translocation, which highlighted its therapeutic potential for zonular fiber reconstruction in vivo.

### 2.6 L‐CN Treatment Promotes the Restoration of Zonular Fibers and Inhibits Inflammatory Infiltration in Ocular Microenvironment in an EL Rat Model

**FIGURE 6:**
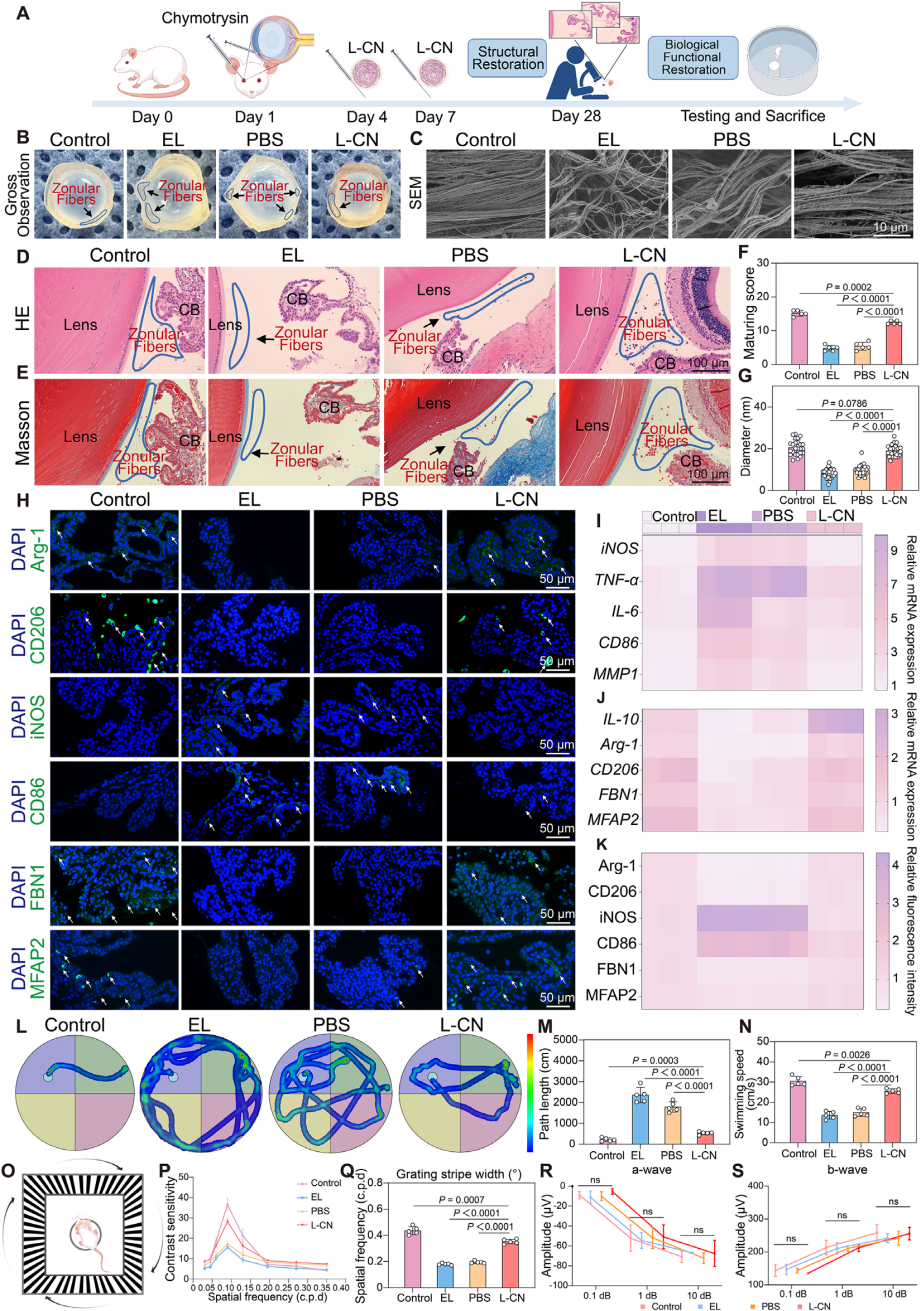
L‐CN treatment offered therapeutic benefits in an EL rat model. (A) Schematic illustration of L‐CN for the treatment of EL rats. (B) Macroscopic images of lens dislocation from different groups. The black arrow points to the zonular fibers. (C) SEM images showing zonular fiber tissues after treatment. Scale bar, 10 µm. (D,E) Results of H&E and Masson’ s staining of zonular fibers. The black arrow points to the zonular fibers. Scale bar, 100 µm. (F) A maturity score of zonular fibers in different groups (*n* = 5). (G) Diameters of zonular fibers from each group (*n* = 25). (I,J) Analysis of genes expression for the zonular fibers and inflammation from the indicated treatment groups after 21 days of treatment (*n* = 3). (H,K) Representative IF staining images of the zonular fibers (white arrow) from the indicated treatment groups after 21 days of treatment and the relative fluorescence intensity (*n* = 3). Scale bar, 50 µm. (L) Swimming trajectories of rats in MWM. (M) Path length (*n* = 5). (N) Swimming speed (*n* = 5). (O) Schematic experimental procedure of the optomotor test. (P,Q) Contrast sensitivity (*n* = 5). (R,S) ERG changes after 21 days of treatments (*n* = 3). Statistical analyses were performed using One‐way ANOVA followed by Tukey's multiple comparisons test (F, G, M, N, Q) and two‐way ANOVA with Dunnett's multiple comparisons test (R,S). Data are presented as mean ± SD. ns, not significant.

To further evaluate the therapeutic efficacy of L‐CN, we established an EL rat model and performed comprehensive in vivo assessments (Figure 6A). Three weeks after treatment, macroscopic observations revealed a substantially reduced area of zonular fiber damage in the L‐CN‐treated group compared to the EL and PBS groups, and a more normal ocular structure was restored (Figure 6B). Scanning electron microscopy (SEM) images demonstrated that the zonular fibers in the L‐CN group exhibited a more organized and well‐aligned architecture, with a significantly greater average fiber diameter (18.95 ± 2.73 nm) than those in the EL (8.05 ± 2.30 nm) and PBS (9.68 ± 2.56 nm) groups (*p* < 0.05) (Figure 6C,G and Figure S12A–D). Hematoxylin‐eosin (H&E) and Masson's trichrome staining further confirmed that L‐CN treatment markedly improved the structural organization of the zonular fibers and reduced inflammatory infiltration, whereas untreated EL and PBS groups showed disorganized fibers (Figure 6D,E). Additionally, based on histological evaluation, a maturity scoring system for the zonular fibers was applied, showing that the L‐CN‐treated group displayed a significant improvement in zonular fiber maturation (12.40 ± 0.55), versus the EL group (4.80 ± 0.84) and PBS group (5.40 ± 1.14) (*p* < 0.05) (Figure 6F and Table S1). These findings collectively suggested that L‐CN effectively promoted the reconstruction of the damaged zonular fibers and restored their structural integrity. To elucidate the mechanisms underlying L‐CN‐mediated repair, we assessed the expression of macrophages polarization markers (M_1_ and M_2_) together with key structural components of the zonular fibers. After three weeks of treatment, RT‐qPCR analysis revealed a significant upregulation of M_2_ macrophage‐associated genes (*IL‐10*, *Arg‐1*, and *CD206*), as well as zonular fiber structural genes (*FBN1* and *MFAP2*), accompanied by suppression of M_1_ macrophage‐associated genes (*p* < 0.05; Figure 6I,J). These results were further confirmed at the protein level through IF staining (Figure 6H,K). Collectively, these findings illustrated that L‐CN treatment not only promoted the structural restoration of zonular fibers but also ameliorated the local inflammatory microenvironment, underscoring its therapeutic potential for zonular fiber repair.

A marked decline in spatial discrimination ability was observed as characteristic visual deficits associated with EL. In the Morris water maze (MWM) test, EL rats exhibited disorganized swimming trajectories, whereas L‐CN treatment substantially improved visual performance (Figure 6L). Both the path length and the escape latency differed significantly among groups (Control: 224.7 ± 86.98 cm, 5.27 ± 0.87 s; EL: 2357 ± 365 cm, 76.82 ± 16.49 s; PBS: 1786 ± 244.3 cm, 65.73 ± 9.45 s; L‐CN: 535.7 ± 72.82 cm, 19.79 ± 6.56 s; Figure 6M and Figure S13A). L‐CN markedly reduced both parameters relative to EL and PBS groups, and the swimming distance was not significantly different from controls (*p* = 0.0674). Furthermore, the average swimming speed was significantly decreased in EL rats (13.60 ± 1.87 cm/s) compared with controls (30.43 ± 2.26 cm/s), while this reduction was restored by L‐CN treatment (25.57 ± 1.11 cm/s; Figure 6N). These findings indicated that the impaired visual function in EL could be rescued by L‐CN treatment (Video S1). Optomotor response testing is widely used in visual prosthesis research to characterize behavioral visual acuity and contrast sensitivity (Figure 6O). Contrast sensitivity was defined as the reciprocal of the minimum detectable grating contrast, whereas visual acuity was determined as the highest spatial frequency of a 100% contrast drifting grating (corresponding to a contrast sensitivity score of 1 in Figure 6P) that the rat was able to track. Normal rats showed a visual acuity of 0.436 ± 0.031 c.p.d. (Figure 6Q). In contrast, EL rats treated with L‐CN achieved a contrast sensitivity score of 15 and a peak response at 0.09 c.p.d. (Figure 6P), with visual acuity recovering to 0.352 ± 0.018 c.p.d. (Figure 6Q), which represented a marked improvement compared with untreated EL rats. In addition, electroretinogram (ERG) recordings showed no significant differences in a‐ and b‐wave amplitudes among the groups before and after 21 days of treatment, demonstrating preserved function of photoreceptors, Müller cells, and retinal pigment epithelium following L‐CN administration (Figure 6R,S and Figure S13B,C).

### 2.7 ScRNA‐seq Analysis Reveals That L‐CN Promotes CD163^+^ M_2_ Macrophages Polarization in EL

**FIGURE 7:**
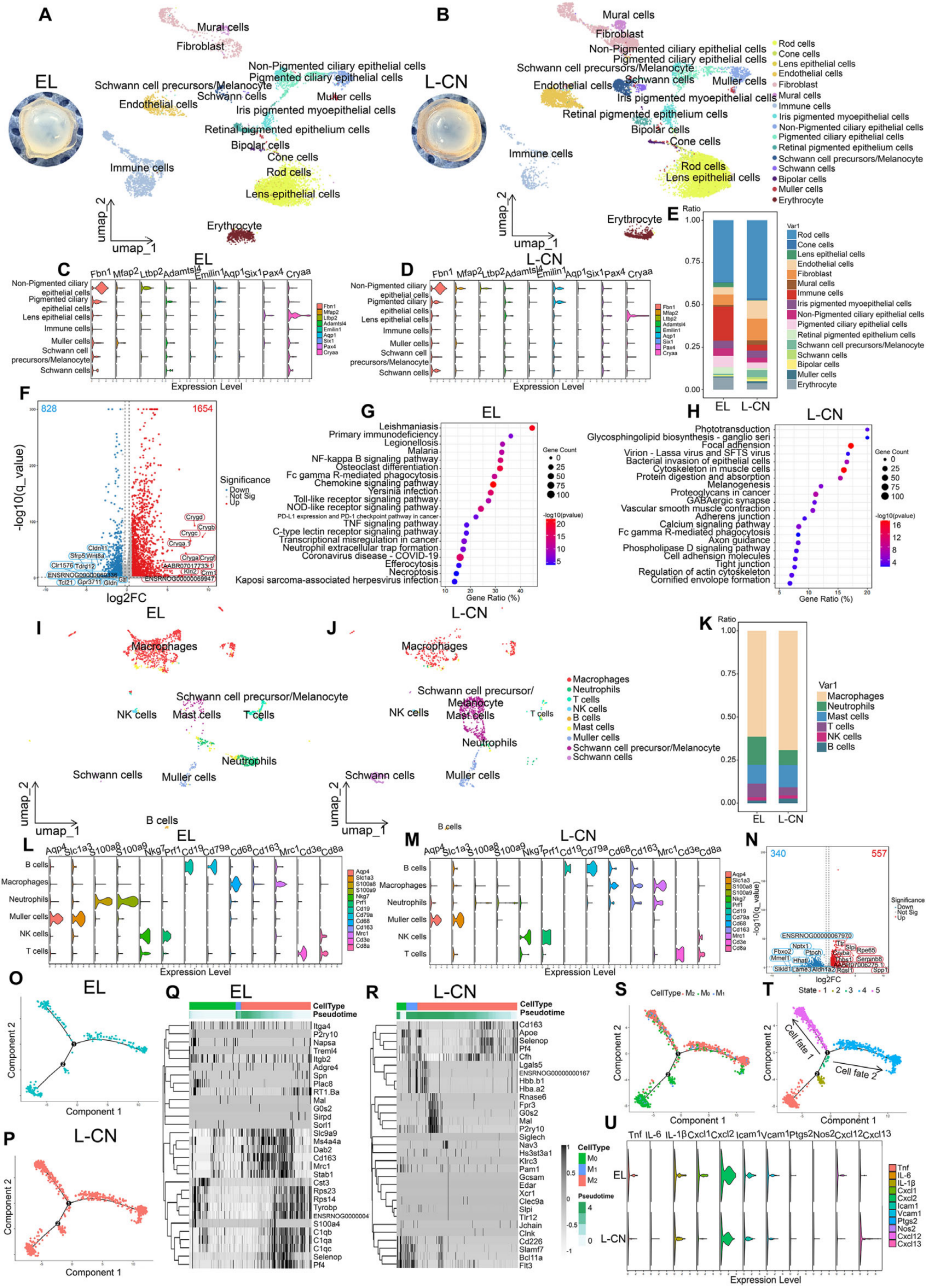
ScRNA‐seq uncovered that L‐CN rescued inflammation in EL rat repairing model. (A,B) General photos and UMAP plots of transcriptional landscape of ciliary body‐zonule complexes from EL and L‐CN groups, colored according to cell types. (C,D) Violin plots of specific gene expressions in selected cells from EL and L‐CN groups. (E) Bar plot of the proportions of 16 major cell clusters in EL and L‐CN groups. (F) Volcano map showing the DEGs of NPCECs between EL and L‐CN groups. (G,H) The top 20 KEGG pathways enriched with the upregulated genes in the EL and L‐CN subgroups. (I,J) Immune cells were re‐clustered into 9 subtypes in the EL and L‐CN groups. (K) Bar plot showing the comparison of the fractions of immune cells subtypes between the EL and L‐CN groups. (L,M) Violin plots of the expression level of selected genes in immune cells of two groups. (N) Volcano map showing the DEGs of immune cells between EL and L‐CN groups. (O,P) The developmental trajectory of macrophages from EL and L‐CN groups according to pseudotime. (Q,R) Expression heatmap of key genes in macrophages of two groups. (S,T) The developmental trajectory of the macrophages from all the samples colored according to subtypes and pseudotime. (U) Expression violin plot of key genes in NF‐κB pathway between EL and L‐CN groups.

To systematically investigate the in vivo targets and regulatory mechanisms of L‐CN, we collected ciliary body‐zonule complex tissues from both the EL model and L‐CN‐ treated group for scRNA‐seq. Through nonlinear dimensionality reduction analysis (UMAP) based on canonical marker genes, we identified and annotated 16 distinct cell populations (Figure 7A,B and Figure S14A,B). Violin plot analysis (Figure 7C,D) revealed a significant reduction in immune‐cell infiltration following L‐CN treatment (3.45% vs. 20.17%; Figure 7E), suggesting that L‐CN effectively alleviated inflammation‐associated cells infiltration. KEGG enrichment analysis of differentially expressed genes showed that, compared to L‐CN ‐treated group, genes upregulated in the EL group were predominantly enriched in classical inflammatory pathways, including cytokine signaling, NF‐κB signaling, TNF signaling, and Toll‐like receptor pathways. In contrast, genes upregulated following L‐CN treatment were significantly enriched in ECM‐related pathways, such as “focal adhesion” and “cytoskeleton in muscle cells” (1654 genes upregulated, 828 genes downregulated; Figure 7F–H and Figure S14C–F), which emphasized the dual functions of L‐CN in promoting both inflammation resolution and ECM remodeling (Figure S14G). We further classified immune cells into nine subpopulations and found that L‐CN treatment significantly reduced the infiltration of T cells (Cd8a, Cd3e; 7.70% vs. 4.83%), neutrophils (S100a9, S100a8; 16.26% vs. 8.70%), and NK cells (Prf1, Nkg7; 2.02% vs. 1.69%), while the proportion of M_2_ macrophages (Mrc1, Cd68) notably increased (61.43% vs. 69.32%; Figure 7I–M and Figure S15A–E). Further subclustering of macrophages into M_0_, M_1_, and M_2_ phenotypes revealed a significant decrease in M_1_ macrophages accompanied by a notable increase in CD163^+^ M_2_ macrophages after L‐CN treatment (Figure S15F–I). Consistent with these findings, pro‐inflammatory mediators, including TNF‐α, Cxcl1, and Cxcl2 were significantly downregulated in L‐CN‐treated group (Figure 7N,U). Additionally, developmental trajectory heatmaps further revealed elevated expression of Itga4 in EL group, a gene that has been reported to be closely associated with M_1_ macrophages migration and inflammation activation [37]. In contrast, CD163 expression was markedly upregulated following L‐CN treatment (Figure 7O–T and Figure S16A–C), supporting its role as a key anti‐inflammatory effector during tissue repair. Conclusively, these findings indicated that L‐CN reduced immune cells infiltration and reshaped macrophage phenotypes within the local microenvironment. In particular, the promotion of CD163^+^ M_2_ macrophages polarization appeared to be a crucial role in facilitating ECM repair and tissue remodeling.

### 2.8 Intravitreal L‐CN Administration Maintains Retinal Function and Exhibits Favorable Biosafety

Comprehensive biosafety evaluations were performed to assess the safety of intravitreal L‐CN administration. Retinal function analysis by optical coherence tomography (OCT) confirmed that retinal structure remained well‐preserved 8 weeks after injection, with no observable structural abnormalities compared to untreated controls (Figure S17A), Additionally, systemic toxicity assessment revealed no histopathological abnormalities in major organs (heart, liver, spleen, lung, and kidney) via H&E staining (Figure S17B). Serum biochemical analyses further indicated that intravitreal injection of L‐CN did not induce significant adverse effects on physiological or metabolic functions (Figure S17C–G). Collectively, these findings demonstrated that L‐CN possessed a favorable safety profile, with no evident local retinal toxicity or systemic side effects after intravitreal administration.

## 3 Discussion

In this study, we first employed an integrated multi‐omics strategy combining bulk RNA‐seq, proteomics, and metabolomics to elucidate the pathological progression of EL, which is characterized by abnormal FBN1 protein structure, excessive ECM degradation, and inflammatory infiltration. Based on these pathological mechanisms, we designed a chiral polymer, L‐CN, through covalent grafting of L‐cys, a naturally occurring chiral amino acid, onto a PAU backbone. L‐CN exhibited potent immunomodulatory activities by inhibiting the phosphorylation and nuclear translocation of NF‐κB in RAW 264.7 cells, thereby suppressing M_1_ macrophages polarization and reducing the release of pro‐inflammatory cytokines. In an in vitro EL cell model, conditioned medium derived from *FBN1*‐silenced HLECs promoted M_1_ macrophages polarization, while this process was effectively attenuated by L‐CN treatment. In an EL rat model, scRNA‐seq revealed that L‐CN significantly enhanced the polarization of CD163^+^ M_2_ macrophages. Histological assessment, SEM imaging, and molecular biological analyses collectively indicated that intravitreal administration of L‐CN effectively promoted both structural and functional recovery of the zonular fibers. This chiral nanoparticle, engineered through a graft‐polymerization strategy, presents a promising therapeutic platform for tissue repair in ECM‐related disorders.

Current surgical intervention, although considered the only effective clinical treatment for EL, is associated with substantial risks, including intraoperative disruption of ocular physiological barriers and high rates of postoperative complications that may ultimately result in vision loss [38, 39]. Increasing evidence has confirmed that the core pathological mechanisms of EL involve dysregulated expression of key zonular proteins, particularly FBN1 and MFAP2, accompanied by aberrant secretion of pro‐inflammatory cytokines (e.g., TNF‐α) and matrix metalloproteinases (MMP‐9/13). These pathological changes collectively contribute to a self‐perpetuating cycle of “ECM degradation—inflammatory infiltration” [13, 22]. Notably, the monocyte‐macrophage system plays a central regulatory role in this process: ECM degradation products can act as damage‐associated molecular patterns, thereby triggering monocytes/macrophages recruitment and promoting M_1_ macrophages polarization, which further aggravates the structural and functional impairment of ECM [40, 41]. This vicious cycle ultimately leads to progressive zonular fiber degeneration and lens dislocation. A deeper understanding of these pathological mechanisms may therefore provide an important theoretical basis for the development of targeted therapeutic strategies.

Based on the above findings, modulation of macrophages polarization appears to be a key factor in promoting zonular fiber repair during EL progression. Increasing evidence has demonstrated that immune regulation plays a central role in tissue regeneration, particularly through the dynamic transition between pro‐inflammatory M_1_ macrophages and reparative M_2_ macrophages. While pro‐inflammatory M_1_ macrophages activation can amplify inflammatory damage and ECM destruction, anti‐inflammatory M_2_ macrophages contribute to inflammation resolution and tissue remodeling [42, 43, 44, 45]. Wang et al. demonstrated that sodium aescinate modulated macrophage polarization balance to control fibrosis and ECM component deposition, therefore treating lymphedema [46]. Similarly, Kang et al. showed that umbilical cord‐derived factors could counteract M_1_‐mediated inflammatory microenvironment to enhance tendon‐bone interface healing [47]. Consistent with these observations, our results suggested that L‐CN effectively interrupted the “ECM degradation—inflammatory infiltration” cycle in EL by suppressing inflammatory activation through the NF‐κB signaling pathway, and promoted a reparative immune microenvironment. Mechanistically, the inhibitory effect of L‐CN on NF‐κB signaling pathway may be associated with both redox regulation and biointerface‐mediated interactions. During polymer degradation, Cys‐derived thiol groups are gradually released and participate in intracellular redox regulation. As an important precursor for glutathione synthesis, Cys is essential for maintaining cellular redox homeostasis. Altered thiol availability may consequently regulate intracellular reactive oxygen species (ROS) levels, which are recognized as upstream modulators of the NF‐κB signaling pathway [48, 49]. Reduced ROS may further suppress IκB phosphorylation and NF‐κB nuclear translocation, thereby attenuating downstream inflammatory signaling pathways. In addition, the chiral biointerface may influence protein adsorption and cell‐material interactions, which further contributes to immune modulation [50, 51]. These combined effects may collectively explain the observed suppression of inflammatory activation and the subsequent preservation of zonular integrity.

Chirality, a fundamental property of biological systems, plays an important role in molecular recognition, cellular interaction, and biological function across multiple scales [52, 53]. Previous studies have shown that chiral nanoparticles could regulate stem cell behavior, immune responses, and tissue regeneration processes [54, 55]. For example, Wei et al. reported that the chirality of a constructed 3D ECM could control mesenchymal stem cell lineage diversification in vitro [56]. These properties have paved the way for the application of chirality for advanced applications in nanomedicine and tissue engineering. Among naturally occurring chiral biomolecules, Cys has attracted considerable attention because it possesses highly reactive amino and thiol groups. These characteristics make it particularly suitable for polymer functionalization [57, 58]. Zhang et al. introduced L‐Cys into gold nanoparticles (L‐Cys‐AuNPs), which promoted periodontal tissue regeneration through autophagy induction [59]. Meanwhile, polyurethanes, which are synthesized through polyaddition reactions between polyols and diisocyanates, have been widely explored owing to their tunable mechanical properties, chemical resistance, and structural flexibility [60]. Inspired by these findings, we grafted L‐Cys onto the PAU side chains to construct a biodegradable chiral polymer system with sustained local bioactivity.

Notably, compared with free small‐molecule therapeutics, polymer‐grafted cysteine may offer distinct advantages for ocular delivery. Free cysteine derivatives are rapidly metabolized and cleared in vivo, which results in short systemic half‐lives. For instance, S‐allyl‐L‐cysteine displays a terminal half‐life of approximately 0.77 h in mice, and N‐acetylcysteine shows a half‐life of 5.6 h following intravenous administration in humans, all of which indicate rapid clearance from circulation [61, 62]. By incorporating L‐Cys into the polymer backbone, the material enables controlled release through gradual degradation, thereby prolonging local retention and reducing the need for repeated intraocular administration [63]. This sustained delivery behavior may be particularly beneficial for ocular diseases, where long‐term local bioactivity and minimized injection frequency are highly desirable. Importantly, although cell membrane receptors such as Toll‐like receptor‌s, CD44, and clathrin are known to participate in immune recognition and nanoparticle internalization [12, 28, 64, 65], our study did not observe significant chirality‐dependent differences in endocytic efficiency among the three nanoparticle groups. Instead, the therapeutic effects appeared to be more closely associated with the sustained release and biological activity of L‐Cys during the degradation of the polymer. These findings suggested that the chiral polymer system primarily functioned through continuous immunomodulatory regulation within the local microenvironment. To our knowledge, this represents the first study to: (1) establish an in vitro EL cell model, (2) employ sc‐RNA seq and multi‐omics to delineate the “ECM degradation—inflammatory infiltration” cycle in EL pathogenesis, and (3) apply chiral polymer therapeutics to ocular disease treatment. Our grafting strategy addresses key translational challenges by enabling sustained therapeutic efficacy without the need for repeated administration, while avoiding the regulatory complexities of drug‐device combinations, thus providing a promising framework for the future development of chiral biomaterials in regenerative medicine and biomedical applications.

Despite the promising findings, there are several limitations in this study. First, flow cytometry and confocal imaging cannot definitively distinguish membrane‐bound nanoparticles from internalized nanoparticles. TEM supports intracellular localization, but quantitative subcellular trafficking and endosomal localization remain unresolved. Future studies employing complementary imaging techniques are needed to further validate nanoparticle internalization and intracellular trafficking. Second, comprehensive assessment of zonular fiber integrity and stability in rodent models remains technically challenging due to anatomical size constraints, highlighting the need for improved biomechanical evaluation methods. Furthermore, the trypsin‐induced model reflects an acute enzymatic injury and thus cannot fully capture the chronic and genetically driven pathophysiology of clinical EL, which warrants further investigation using more representative disease models. Finally, although the investigation focused primarily on the spatiotemporal dynamics of monocytes and macrophages, sc‐RNA seq also revealed the involvement of additional immune cell populations, including neutrophils, T cells, and B cells. Future studies will further explore the interactions between monocytes/macrophages and other immune cells.

## 4 Conclusion

In conclusion, our study provides new insights into the core pathological mechanism underlying EL, namely the vicious cycle of ECM degradation and inflammatory infiltration, and presents an innovative chiral polymer, L‐CN, which developed through a grafting‐based strategy. By incorporating L‐Cys onto the copolymer side chains, L‐CN exhibited dual immunomodulatory effects by suppressing M_1_ macrophages polarization while promoting the secretion of anti‐inflammatory factors from M_2_ macrophages. As the polymer backbone gradually degraded, L‐CN effectively regulated ECM remodeling through potent inhibition of NF‐κB signaling pathway, thereby preserving the expression of critical zonular proteins FBN1 and MFAP2. In vivo studies further demonstrated that three weeks of L‐CN treatment significantly increased the accumulation of CD163^+^ M_2_ macrophages (533 vs. 186 in the EL group), attenuated inflammatory responses, and disrupted the pathological cycle, ultimately facilitating the restoration of zonular fiber structural integrity. The demonstrated efficacy of L‐CN in EL treatment suggests its broader applicability to other ECM‐related pathologies, such as osteoarthritis and cardiovascular disorders, in which chronic inflammation and excessive MMP‐mediated ECM degradation are central pathological features. The dual ability of L‐CN to suppress inflammatory signaling pathways while preserving collagen and proteoglycan integrity may therefore enable targeting the fundamental mechanisms driving ECM deterioration [66, 67]. Collectively, this study establishes a scalable platform for immunomodulation‐driven tissue construction and underscores the considerable translational potential of chiral nanoparticles as next‐generation therapeutic nanomaterials for ophthalmic diseases and regenerative medicine.

## 5 Experimental Section/Methods

### 5.1 Patient Selection for EL Cohort

This study was approved by the Human Research Ethics Committee of the Eye and ENT Hospital of Fudan University (ChiCTR2000039132) and conducted in accordance with the Declaration of Helsinki. Patients diagnosed with congenital EL were consecutively enrolled during clinical visits between July 2023 and April 2025. Inclusion criteria were as follows: diagnosis of incomplete EL or spherophakia confirmed via slit‐lamp examination under full mydriasis, availability of comprehensive medical records, and consent to undergo genetic testing and subsequent follow‐up evaluations. Exclusion criteria included a history of prior intraocular surgery, ocular trauma, or concomitant ocular diseases such as corneal dystrophy, retinal pigmentary degeneration, retinal detachment, or glaucoma [68, 69, 70].

### 5.2 Control Cohort Selection

Control participants were selected from CC patients treated at the same institution during the same period. Eligibility criteria included: absence of EL or spherophakia confirmed by dilated slit‐lamp examination, availability of complete medical records, and consent to genetic testing and follow‐up. Exclusion criteria were identical to those of the EL cohort to ensure comparability between the groups.

### 5.3 Panel‐Based Next‐Generation Sequencing and Multi‐Omics Analysis

Peripheral blood samples from 100 EL patients were collected for genetic analysis, and sequencing was performed by We‐health Technology Co., Ltd (Shanghai, China). Mutation sites in the *FBN1* gene were visualized using a Lollipop Chart generated with SnapGene software (version 7.2). Anterior lens capsular tissue samples were obtained from 35 EL patients and 30 CC subjects during cataract surgery. RNA‐seq was performed by Fudan University. The remaining tissue samples were divided for RT‐qPCR validation (Table S2) and IF staining to evaluate gene expression and protein localization. Aqueous humor samples (150 µL per patient) were collected from 5 EL and 5 CC patients during surgery, immediately snap‐frozen in liquid nitrogen, and stored at −80°C until analysis. Aqueous humor protein microarray analysis was performed by Shanghai Yuling Company. Metabolites were extracted using a direct deproteination technique and analyzed by Fudan University.

### 5.4 Synthesis and Biocompatibility Evaluation of PAU‐g‐L‐Cys and L‐CN

Poly (ester‐dimethylolbutanoic acid) urethane‐graft‐L‐Cys (PAU‐g‐L‐Cys) was synthesized, with D‐configuration PAU‐g‐D‐Cys and racemic PAU‐g‐DL‐Cys elastomers prepared using D‐Cys and DL‐Cys, respectively. The chemical structures of the products were characterized by ^1^H‐NMR (using DMSO‐d6 as solvent) and FTIR with an attenuated total reflection accessory. L‐configuration PAU‐g‐L‐Cys nanoparticles (L‐CN) were then prepared, along with D‐configuration PAU‐g‐D‐Cys nanoparticles (D‐CN) and DL‐configuration PAU‐g‐DL‐Cys nanoparticles (DL‐CN) following the same protocol. The nanoparticles were characterized by TEM for morphology, DLS for hydrodynamic size and zeta potential, and CD spectroscopy to confirm their chiral structure. The stability of L‐CN was assessed over a one‐week period in PBS at 25°Cand 37°C (pH 7.1, pH 7.4, and pH 7.5) by monitoring changes in particle size using DLS. Cytotoxicity of D‐CN, L‐CN, and DL‐CN was evaluated using a CCK‐8 kit (Beyotime, Shanghai, China), a Viability/Cytotoxicity Kit (Beyotime, Shanghai, China), and flow cytometry.

### 5.5 Primary Culture of BMDMs and HLECs

BMDMs were isolated from 8‐week‐old C57BL/6J mice in accordance with approved ethical guidelines. HLECs were purchased from ZQXZBIO (Shanghai, China) and cultured in α‐minimum essential medium (α‐MEM; Gibco, USA) supplemented with 10% fetal bovine serum (FBS; Gibco) and 1% penicillin‐streptomycin (complete α‐MEM) in a humidified incubator at 37°C and 5% CO_2_. The culture medium was refreshed every 3 days. After 2–3 passages, HLECs were used for wound healing assay, transwell migration assay and subsequent experiments.

### 5.6 in vitro Phagocytosis Assay and in vivo Biodistribution of D‐CN, L‐CN, and DL‐CN

The cellular uptake efficiencies of D‐CN, L‐CN and DL‐CN were evaluated using a multi‐modal approach combining IF staining, flow cytometry and TEM analysis in RAW 264.7 cells. Nanoparticle biodistribution in ocular tissues was dynamically monitored using an in vivo imaging system to assess their pharmacokinetic profiles and retention characteristics. The nanoparticles were labeled with FITC and Cy7 according to the manufacturer's protocols.

### 5.7 Immunomodulatory Effects of D‐CN, L‐CN, and DL‐CN

Macrophages polarization toward M_1_ and M_2_ phenotypes was induced in the presence or absence of D‐CN, L‐CN, and DL‐CN. After 48 h of incubation, cells were collected for RT‐qPCR, IF staining, WB, and flow cytometry analyses. Total RNA was extracted using TRIzol reagent (Invitrogen, Carlsbad, CA, USA) and submitted to LC‐Bio Technology Co., Ltd. (Hangzhou, China) for RNA‐seq analysis.

### 5.8 Establishment and Evaluation of Cell Models of EL

HLECs were seeded into 6‐well plates and allowed to adhere for 24 h prior to transfection. SiRNA targeting *FBN1* in HLECs was purchased from Genomeditech (Shanghai, China) and transfected using Lipofectamine 2000 (Thermo Fisher Scientific, USA) according to the manufacturer's protocol. After 48 h of incubation, cells or cell lysates were collected for analysis. RT‐qPCR, IF staining, and WB were performed to verify the knockdown efficiency of *FBN1*. Total RNA was extracted using TRIzol reagent (Invitrogen, Carlsbad, CA, USA) and submitted to LC‐Bio Technology Co., Ltd. (Hangzhou, China) for RNA‐seq.

### 5.9 Regulation of NF‐κB Signaling Pathway

RAW 264.7 cells were seeded into 6‐well plates and cultured under the following conditions: (1) negative control—high‐glucose Dulbecco's modified Eagle's medium (H‐DMEM; Gibco, USA) supplemented with 10% FBS and 1% penicillin‐streptomycin (complete DMEM) alone; (2) positive control—complete DMEM with 200 ng/mL lipopolysaccharide (LPS; Sigma, USA); and (3) experimental groups—complete DMEM containing 200 ng/mL LPS together with D‐CN, L‐CN, or DL‐CN. After 24 h of incubation, cells were harvested for IF staining and WB, and supernatants were collected for ELISA.

### 5.10 Establishment, Histology and Immunohistochemical Analysis of L‐CN Repair EL Model

This study was approved by the Institutional Animal Care and Use Committee (IACUC) of the Eye & ENT Hospital, Fudan University (IACUC‐DWZX‐2025‐042). All animal procedures were performed in accordance with institutional and national guidelines for the care and use of laboratory animals, and were designed to minimize animal suffering and reduce the number of animals used. The EL model was established according to the protocol. After successful modeling, 8 µL of either PBS or 5 mg/mL L‐CN solution was injected into the vitreous body of the PBS and L‐CN groups. The EL group received no intervention. Treatments were administered two doses following initial modeling (q3d). At 21 days after treatment, rats were sacrificed, and the intact ciliary body‐zonule complex tissue was harvested for histological examination, ultrastructural analysis, RT‐qPCR, IF staining, and scRNA‐seq. Structural and morphological analyses were conducted by gross observation and scanning electron microscope (SEM, RISE‐MANGA, Czech Republic) at 15.0 kV. H&E staining and Masson's trichrome staining were performed according to standard protocols for histological analysis. Fiber maturing scores were evaluated based on H&E and Masson staining, and the higher scores indicated improved zonular fiber reconstruction.

### 5.11 Single‐Cell RNA Sequencing

Ciliary body‐zonule complex tissues were harvested from different groups under a surgical microscope and pooled to obtain sufficient cell numbers for analysis. ScRNA‐seq was performed by MobiDrop Co., Ltd (Zhejiang, China). using the Chip A Single Cell Kit v2.1 (MobiDrop, S050100301) according to the manufacturer's instructions.

### 5.12 Ocular Biocompatibility Assay

Ocular safety following intravitreal injection was evaluated using OCT (ISOCT‐II, Optoprobe) and histopathological analysis. At 8 weeks post‐injection, peripheral blood was collected via cardiac puncture under inhalation anesthesia for hematological and biochemical assessments, including complete blood count and serum biochemistry analyses. Rats were subsequently euthanized for histopathological examination. Tissue sections (5 µm) were stained with H&E to evaluate tissue morphology and cellular integrity.

### 5.13 Behavioral Tests

Three weeks after injection, EL rats underwent MWM, optomotor response, and ERG tests to evaluate visual acuity and contrast sensitivity. All experimental procedures were performed in accordance with established protocols.

### 5.14 Statistical Analysis

Analyses were performed using GraphPad Prism 9.5.0 software and R (version 2024.4.4.1), and all data are presented as mean ± standard deviation (mean ± SD). Transcriptional profiling was analyzed using publicly available bioinformatics platforms. Sample sizes commonly used in rats’ studies were adopted, and the exact number of biological replicates or animals included in each analysis is indicated in the corresponding figure legends. For comparisons between two groups with normally distributed data, an unpaired two‐tailed Student's t‐test was used, while paired two‐tailed Student's t‐tests were applied for matched samples. Comparisons among multiple groups were analyzed using one‐way ANOVA followed by Tukey's post hoc test. Non‐normally distributed data were analyzed using the Mann‐Whitney U test or Kruskal‐Wallis test with Dunn's multiple comparisons test. For experiments involving two independent variables, two‐way ANOVA followed by Dunnett's multiple comparisons test was performed. All statistical analyses were conducted using two‐tailed tests unless otherwise specified, and a *p* value < 0.05 was considered statistically significant. For animal experiments, sample sizes were determined based on previous experimental experience and statistical power considerations. All studies included sufficient numbers of animals to ensure reliable statistical analysis, and the exact sample sizes are provided in the corresponding figures or legends. Animals were randomly assigned to experimental groups whenever possible, and investigators were blinded to group allocation during data collection and outcome assessment. Representative images shown in the manuscript were selected from repeated independent experiments and were chosen based on their consistency with the overall quantitative results.

## Author Contributions

Y.W., Y.Y., H.G., Y.L. and X.W. contributed equally to this work. Y.J., T.C., and L.W. conceived the study. Y.W., T.C., L.W., and Y.L. wrote the paper. R.L. and Y.W. collected the ocular tissue and aqueous humor of patients from Y.J. and Z.Z., with multi‐omics analysis help from L.S. Y.L., Y.Y., and H.G. synthesized and conducted characterization tests of L‐CN, D‐CN and DL‐CN. Y.W. carried out the safety evaluation of three chiral nanoparticles, together with in vitro anti‐inflammatory assays and mechanistic studies, refined the techniques and analyzed the data, with help from Y.L., H.G., X.W, A.M., J.X., S. J., and L.L. Y.W., Y.L., and X.W. established the EL rat models and performed both in vivo and in vitro phenotype restoration assays. Y.W. and Y.L. also analyzed the single‐cell sequencing analysis with help from L.S. and D.L. Y.W., L.Y., X.W., and L.S. performed rats’ behavioral assays and safety studies with help from T.C. and D.L. W.Y., T.C., and L.W. analyzed the original data. All authors discussed the results and commented on the manuscript. All authors contributed to the manuscript and overall discussion.

## Funding

This work was supported by the National Natural Science Foundation of China (grant nos. 82571187, 82271068, and 82401230) to Y.J.; the National Natural Science Foundation of China (grant no. 82502973) to L.W.; the China Postdoctoral Science Foundation (grant no. 2023M742342) to L.W.; the Shanghai Sailing Program (grant no. 24YF2701700) to L.W.; the 74th Postdoctoral Science Foundation of China (No. 2023M742342 to L. W.); the Foundation of National Facility for Translational Medicine (Shanghai) (TMSK‐2024‐202) to L.W.; the Foundation of National Center for Translational Medicine (Shanghai) SHU Branch (SUITM‐202512) to L.W.; Shanghai Oriental Talent Plan (No. QNWS2025073 to L. W.); Shanghai Sixth People's Hospital (No. ynqn202506 to L. W.); the Open Research Project of Shanghai Key Laboratory of Rare Disease Gene Editing and Cell Therapy (gect: ‐2025‐Q06) to T.C.

## Conflicts of Interest

The authors declare no conflict of interest.

## Supporting information




**Supporting File 1**: advs76795‐sup‐0001‐SuppMat.docx.

**Supporting File 2**: advs76795‐sup‐0002‐VideoS1.mp4.

## Data Availability

The datasets generated and/or analyzed during the current study are available from the corresponding author on reasonable request.

## References

[1] M. Liu , L. Zhang , and T. Wang , “Supramolecular Chirality in Self‐Assembled Systems,” Chemical Reviews 115, no. 15 (2015): 7304–7397, 10.1021/cr500671p. 26189453

[2] X. Dou , N. Mehwish , C. Zhao , J. Liu , C. Xing , and C. Feng , “Supramolecular Hydrogels with Tunable Chirality for Promising Biomedical Applications,” Accounts of Chemical Research 53, no. 4 (2020): 852–862, 10.1021/acs.accounts.0c00012. 32216333

[3] J. Li , P. Li , M. Fan , X. Zheng , J. Guan , and M. Yin , “Chirality of Perylene Diimides: Design Strategies and Applications,” Angewandte Chemie (International Ed in English) 61, no. 27 (2022): 202202532, 10.1002/anie.202202532. 35357065

[4] T. Gibaud , E. Barry , M. J. Zakhary , et al., “Reconfigurable Self‐Assembly through Chiral Control of Interfacial Tension,” Nature 481, no. 7381 (2012): 348–351, 10.1038/nature10769. 22217941

[5] H. Jiang , R. Liu , L. Wang , et al., “Chiral‐Selective Antigen‐Presentation by Supramolecular Chiral Polymer Micelles,” Advanced Materials 35, no. 5 (2023): 2208157, 10.1002/adma.202208157. 36398497

[6] X. Luo , S. Zhao , T. Wang , et al., ““Bioactive” Therapeutic Contact Lens Triggered by Biomimetic Chiral Helical Nanoarchitectonics for Rapid Corneal Repair,” ACS Nano 19, no. 9 (2025): 9250–9264, 10.1021/acsnano.5c00298. 39999297

[7] C. Xing , H. Zhu , X. Dou , et al., “Infected Diabetic Wound Regeneration Using Peptide‐Modified Chiral Dressing to Target Revascularization,” ACS Nano 17, no. 7 (2023): 6275–6291, 10.1021/acsnano.2c10039. 36946387

[8] Y. He , X. Zhang , F. Meng , et al., “Biomimetic Chiral Nanotopography for Manipulating Immunological Response,” Advanced Functional Materials 34, no. 12 (2024): 2313157, 10.1002/adfm.202313157.

[9] H. Zhao , J. Jiao , Z. Wang , et al., “Amino Acid Directed Helical Assemblies of Natural Berberine for Chirality‐Dependent Photodynamic Antibacterial Therapy,” Advanced Science 13, no. 3 (2026): 14096, 10.1002/advs.202514096. PMC12806354 41255251

[10] J. Hao , Y. Tian , J. Tang , et al., “Enantiomer‐Dependent Uptake of Chiral Nanoparticles in Macrophages Modulates the Inflammatory Response through the NF‐κB Pathway,” Environmental Science & Technology 59, no. 13 (2025): 6428–6439, 10.1021/acs.est.4c12577. 40126054

[11] M. Xu , W. Xin , J. Xu , et al., “Biosilicification‐Mimicking Chiral Nanostructures for Targeted Treatment of Inflammatory Bowel Disease,” Nature Communications 16, no. 1 (2025): 2551, 10.1038/s41467-025-57890-8. PMC11910640 40089457

[12] J. Luo , B. Shi , C. Hao , et al., “Chiral Zinc Sulfide Nanoparticles Scavenging Reactive Oxygen Species for Remodeling Intestinal Homeostasis,” Angewandte Chemie International Edition 64, no. 23 (2025): 202503654, 10.1002/anie.202503654. 40170506

[13] T. Chen , Z. Chen , J. Du , et al., “Reprogramming of iPSCs to NPCEC‐Like Cells by Biomimetic Scaffolds for Zonular Fiber Reconstruction,” Bioactive Materials 45 (2025): 446–458, 10.1016/j.bioactmat.2024.11.031. 39697240 PMC11653162

[14] D. V. Patel and C. N. J. McGhee , “Hanging by Threads: Ectopia Lentis,” Lancet 384, no. 9946 (2014): 893, 10.1016/S0140-6736(13)61838-3. 24726475

[15] T. Chen , M. Deng , M. Zhang , J. Chen , Z. Chen , and Y. Jiang , “Visual Outcomes of Lens Subluxation Surgery with Cionni Modified Capsular Tension Rings in Marfan Syndrome,” Scientific Reports 11, no. 1 (2021): 2994, 10.1038/s41598-021-82586-6. 33542371 PMC7862488

[16] D. J. Salchow and P. Gehle , “Ocular Manifestations of Marfan Syndrome in Children and Adolescents,” European Journal of Ophthalmology 29, no. 1 (2019): 38–43, 10.1177/1120672118761333. 29587526

[17] J. A. N. Meester , A. Verstraeten , D. Schepers , M. Alaerts , L. V. Laer , and B. L. Loeys , “Differences in Manifestations of Marfan Syndrome, Ehlers‐Danlos Syndrome, and Loeys‐Dietz Syndrome,” Annals of Cardiothoracic Surgery 6, no. 6 (2017): 582–594, 10.21037/acs.2017.11.03. 29270370 PMC5721110

[18] Z. Chen , T. Chen , M. Zhang , et al., “Fibrillin‐1 Gene Mutations in a Chinese Cohort with Congenital Ectopia Lentis: Spectrum and Genotype–phenotype Analysis,” British Journal of Ophthalmology 106, no. 12 (2022): 1655–1661, 10.1136/bjophthalmol-2021-319084. 34281902 PMC9685704

[19] J. Halper and M. Kjaer , “Basic Components of Connective Tissues and Extracellular Matrix: Elastin, Fibrillin, Fibulins, Fibrinogen, Fibronectin, Laminin, Tenascins and Thrombospondins,” in Progress in Heritable Soft Connective Tissue Diseases, ed. J. Halper (Springer, 2014): 31–47, 10.1007/978-94-007-7893-1_3. 24443019

[20] T. Sharma , L. Gopal , M. P. Shanmugam , et al., “Retinal Detachment in Marfan Syndrome: Clinical Characteristics and Surgical Outcome,” Retina 22, no. 4 (2002): 423–428. 12172108 10.1097/00006982-200208000-00005

[21] I. H. Maumenee , “The Eye in the Marfan Syndrome,” Transactions of the American Ophthalmological Society 79 (1981): 684–733. 7043871 PMC1312201

[22] Y. Shi , J. Chen , L. Cai , et al., “Uncovering the Hidden World of Aqueous Humor Proteins for Discovery of Biomarkers for Marfan Syndrome,” Advanced Science 11, no. 6 (2024): 2303161, 10.1002/advs.202303161. 38088571 PMC10853735

[23] A. De Maria , P. A. Wilmarth , L. L. David , and S. Bassnett , “Proteomic Analysis of the Bovine and Human Ciliary Zonule,” Investigative Ophthalmology & Visual Science 58, no. 1 (2017): 573–585, 10.1167/iovs.16-20866. 28125844 PMC5283081

[24] C. Chipeta , J. Aragon‐Martin , and A. Chandra , “Zonulopathies as Genetic Disorders of the Extracellular Matrix,” Genes 15, no. 12 (2024): 1632, 10.3390/genes15121632. 39766898 PMC11675282

[25] A. de la Fuente‐Alonso , M. Toral , A. Alfayate , et al., “Aortic Disease in Marfan Syndrome Is Caused by Overactivation of sGC‐PRKG Signaling by NO,” Nature Communications 12 (2021): 2628, 10.1038/s41467-021-22933-3. PMC8113458 33976159

[26] M. J. Ruiz‐Rodríguez , J. Oller , S. Martínez‐Martínez , et al., “Versican Accumulation Drives Nos_2_ Induction and Aortic Disease in Marfan Syndrome via Akt Activation,” EMBO Molecular Medicine 16, no. 1 (2024): 132–157, 10.1038/s44321-023-00009-7. 38177536 PMC10897446

[27] S. Li , Y. Zhao , J. Chen , et al., “Chirality Regulates Stem Cell Fate and Promotes Corneal Epithelial Regeneration via Manipulating Notch Pathway,” Advanced Science 12, no. 31 (2025): 04732, 10.1002/advs.202504732. PMC12376501 40444446

[28] Z. Li , A. Qu , C. Xu , H. Kuang , L. Xu , and M. Sun , “Chiral Aluminum Oxyhydroxide Supraparticles as Adjuvants,” Advanced Materials 37, no. 29 (2025): 2504458, 10.1002/adma.202504458. 40237037

[29] Y. Chu , W. Zhang , D. Liu , et al., “Ligament Inspired Ultra‐Strong and Tough Bio‐Based Polyurethane Elastomers via Dynamic Hydrogen Bonding Induced Confinement Effect,” Advanced Functional Materials 36, no. 44 (2026): 10461, 10.1002/adfm.202510461.

[30] C. Chu , W. Sun , S. Chen , et al., “Squid‐Inspired Anti‐Salt Skin‐Like Elastomers with Superhigh Damage Resistance for Aquatic Soft Robots,” Advanced Materials 36, no. 44 (2024): 2406480, 10.1002/adma.202406480. 39267419

[31] Q. Liu , P. Lou , Z. Sun , et al., “Bio‐Based Elastomers: Design, Properties, and Biomedical Applications,” Advanced Materials 37, no. 22 (2025): 2417193, 10.1002/adma.202417193. 39801219

[32] P. Song and H. Wang , “High‐Performance Polymeric Materials through Hydrogen‐Bond Cross‐Linking,” Advanced Materials 32, no. 18 (2020): 1901244, 10.1002/adma.201901244. 31215093

[33] M. Dubald , S. Bourgeois , V. Andrieu , and H. Fessi , “Ophthalmic Drug Delivery Systems for Antibiotherapy—A Review,” Pharmaceutics 10, no. 1 (2018): 10, 10.3390/pharmaceutics10010010. 29342879 PMC5874823

[34] N. J. V. Haeringen , “Clinical Biochemistry of Tears,” Survey of Ophthalmology 26, no. 2 (1981): 84–96, 10.1016/0039-6257(81)90145-4. 7034254

[35] S. Mullany , H. Marshall , T. Zhou , et al., “RNA Sequencing of Lens Capsular Epithelium Implicates Novel Pathways in Pseudoexfoliation Syndrome,” Investigative Opthalmology & Visual Science 63, no. 3 (2022): 26, 10.1167/iovs.63.3.26. PMC8982629 35348588

[36] M. A. W. ElKhatib , F. A. Isse , and A. O. S. El‐Kadi , “Effect of Inflammation on Cytochrome P450‐Mediated Arachidonic Acid Metabolism and the Consequences on Cardiac Hypertrophy,” Drug Metabolism Reviews 55, no. 1‐2 (2023): 50–74, 10.1080/03602532.2022.2162075. 36573379

[37] T. Fang , X. Yin , Y. Wang , H. Wang , X. Wang , and Y. Xue , “Lymph Node Metastasis‐Related Gene ITGA4 Promotes the Proliferation, Migration, and Invasion of Gastric Cancer Cells by Regulating Tumor Immune Microenvironment,” Journal of Oncology 2022 (2022): 1–13, 10.1155/2022/1315677. PMC9569201 36254221

[38] K. NB , P. Kohli , B. P. S. Pangtey , and K. Ramasamy , “Evaluation of Sutureless, Glueless, Flapless, Intrascleral Fixated Posterior Chamber Intraocular Lens in Children with Ectopia Lentis,” Journal of Ophthalmology 2018 (2018): 1–6, 10.1155/2018/3212740. PMC6136496 30228913

[39] P. Sen , Y. Attiku , P. Bhende , E. Rishi , D. Ratra , and K. Sreelakshmi , “Outcome of Sutured Scleral Fixated Intraocular Lens in Marfan Syndrome in Pediatric Eyes,” International Ophthalmology 40, no. 6 (2020): 1531–1538, 10.1007/s10792-020-01322-7. 32107694

[40] R. M. Zhang , K. Tiedemann , M. L. Muthu , et al., “Fibrillin‐1‐Regulated miR‐122 Has a Critical Role in Thoracic Aortic Aneurysm Formation,” Cellular and Molecular Life Sciences 79, no. 6 (2022): 314, 10.1007/s00018-022-04337-8. 35606547 PMC11072253

[41] F. D'Amico , E. Doldo , C. Pisano , et al., “Specific miRNA and Gene Deregulation Characterize the Increased Angiogenic Remodeling of Thoracic Aneurysmatic Aortopathy in Marfan Syndrome,” International Journal of Molecular Sciences 21, no. 18 (2020): 6886, 10.3390/ijms21186886. 32961817 PMC7555983

[42] M. C. Bosco , “Macrophage Polarization: Reaching across the Aisle?,” Journal of Allergy and Clinical Immunology 143, no. 4 (2019): 1348–1350, 10.1016/j.jaci.2018.12.995. 30639344

[43] K. B. Sugg , J. Lubardic , J. P. Gumucio , and C. L. Mendias , “Changes in Macrophage Phenotype and Induction of Epithelial‐to‐Mesenchymal Transition Genes Following Acute Achilles Tenotomy and Repair,” Journal of Orthopaedic Research : Official Publication of the Orthopaedic Research Society 32, no. 7 (2014): 944–951, 10.1002/jor.22624. 24700411 PMC4086481

[44] J. Y. Sunwoo , C. D. Eliasberg , C. B. Carballo , and S. A. Rodeo , “The Role of the Macrophage in Tendinopathy and Tendon Healing,” Journal of Orthopaedic Research 38, no. 8 (2020): 1666–1675, 10.1002/jor.24667. 32190920

[45] F. Guan , R. Wang , Z. Yi , et al., “Tissue Macrophages: Origin, Heterogenity, Biological Functions, Diseases and Therapeutic Targets,” Signal Transduction and Targeted Therapy 10, no. 1 (2025): 93, 10.1038/s41392-025-02124-y. 40055311 PMC11889221

[46] X. Wang , C. Cui , X. Meng , et al., “Chiral Supramolecular Hydrogel Enhanced Transdermal Delivery of Sodium Aescinate to Modulate M1 Macrophage Polarization against Lymphedema,” Advanced Science 11, no. 5 (2023): 2303495, 10.1002/advs.202303495. 38037850 PMC10837362

[47] Y. Kang , L. Wang , S. Zhang , et al., “Bioactive Patch for Rotator Cuff Repairing via Enhancing Tendon‐to‐Bone Healing: A Large Animal Study and Short‐Term Outcome of a Clinical Trial,” Advanced Science 11, no. 31 (2024): 2308443, 10.1002/advs.202308443. 38922803 PMC11336973

[48] M. J. Morgan and Z. Liu , “Crosstalk of Reactive Oxygen Species and NF‐κB Signaling,” Cell Research 21, no. 1 (2011): 103–115, 10.1038/cr.2010.178. 21187859 PMC3193400

[49] A. J. Meyer and R. Hell , “Glutathione Homeostasis and Redox‐regulation by Sulfhydryl Groups,” Photosynthesis Research 86, no. 3 (2005): 435–457, 10.1007/s11120-005-8425-1. 16315075

[50] X. Niu , R. Zhao , S. Yan , et al., “Chiral Materials: Progress, Applications, and Prospects,” Small 19, no. 38 (2023): 2303059, 10.1002/smll.202303059. 37217989

[51] S. Han , L. Gao , X. Dou , et al., “Chiral Hydrogel Nerve Conduit Boosts Peripheral Nerve Regeneration via Regulation of Schwann Cell Reprogramming,” ACS Nano 18, no. 41 (2024): 28358–28370, 10.1021/acsnano.4c10653. 39403973

[52] H. Wu , C. Xing , B. Yu , et al., “Metabolic Reprogramming of Neural Stem Cells by Chiral Nanofiber for Spinal Cord Injury,” ACS Nano 19, no. 4 (2025): 4785–4801, 10.1021/acsnano.4c15770. 39841801 PMC11803919

[53] F. Gao , X. Qiu , S. Baddi , et al., “Chiral Nanofibers of Camptothecin Trigger Pyroptosis for Enhanced Immunotherapy,” Angewandte Chemie International Edition 64, no. 13 (2025): 202423446, 10.1002/anie.202423446. 39803865

[54] X. Lv , Y. Tian , F. Wu , et al., “Chiral Plasmonic‐Dielectric Coupling Enables Strong Near‐Infrared Chiroptical Responses from Helicoidal Core‐Shell Nanoparticles,” Nature Communications 15, no. 1 (2024): 9234, 10.1038/s41467-024-53705-4. PMC11511876 39455559

[55] J. Karst , N. H. Cho , H. Kim , et al., “Chiral Scatterometry on Chemically Synthesized Single Plasmonic Nanoparticles,” ACS Nano 13, no. 8 (2019): 8659–8668, 10.1021/acsnano.9b04046. 31294546

[56] Y. Wei , S. Jiang , M. Si , et al., “Chirality Controls Mesenchymal Stem Cell Lineage Diversification through Mechanoresponses,” Advanced Materials 31, no. 16 (2019): 1900582, 10.1002/adma.201900582. 30838715

[57] M. Cazzola , L. Calzetta , F. Facciolo , P. Rogliani , and M. G. Matera , “Pharmacological Investigation on the Anti‐Oxidant and Anti‐Inflammatory Activity of N‐acetylcysteine in an Ex Vivo Model of COPD Exacerbation,” Respiratory Research 18, no. 1 (2017): 26, 10.1186/s12931-016-0500-y. 28118826 PMC5260037

[58] B. Thirumalraj , N. Dhenadhayalan , S.‐M. Chen , et al., “Highly Sensitive Fluorogenic Sensing of L‐Cysteine in Live Cells Using Gelatin‐Stabilized Gold Nanoparticles Decorated Graphene Nanosheets,” Sensors and Actuators B, Chemical 259 (2018): 339–346, 10.1016/j.snb.2017.12.028. 32288250 PMC7127153

[59] S. Zhang , H. Zhou , N. Kong , et al., “L‐Cysteine‐Modified Chiral Gold Nanoparticles Promote Periodontal Tissue Regeneration,” Bioactive Materials 6, no. 10 (2021): 3288–3299, 10.1016/j.bioactmat.2021.02.035. 33778205 PMC7970259

[60] L. Wang , J. Xiang , H. Fan , and Z. Sun , “Hydrophobic High‐Bio‐Based Content Waterborne Polyurethane Prepared by Diols and High‐Molecular Weight Internal Emulsifier,” Collagen and Leather 7, no. 1 (2025): 35, 10.1186/s42825-025-00214-9.

[61] S. A. Sahasrabudhe , M. R. Terluk , and R. V. Kartha , “N‐Acetylcysteine Pharmacology and Applications in Rare Diseases—Repurposing an Old Antioxidant,” Antioxidants 12, no. 7 (2023): 1316, 10.3390/antiox12071316. 37507857 PMC10376274

[62] M. Nakamoto , K. Kunimura , and M. Ohtani , “Pharmacokinetics of Sulfur‑Containing Compounds in Aged Garlic Extract: S‑Allylcysteine, S‑1‑Propenylcysteine, S‑Methylcysteine, S‑Allylmercaptocysteine and Others (Review),” Experimental and Therapeutic Medicine 29, no. 5 (2025): 1–8, 10.3892/etm.2025.12852. 40171136 PMC11959343

[63] C. Xu , Y.‐T. Lu , B. Yang , Z. Li , and K. Zeng , “Recent Progress on Stimuli‐Responsive Chitosan‐Based Biomaterials as Drug Carriers,” Interdisciplinary Medicine 4, no. 3 (2026): 70112, 10.1002/inmd.70112.

[64] K. A. Fitzgerald and J. C. Kagan , “Toll‐Like Receptors and the Control of Immunity,” Cell 180, no. 6 (2020): 1044–1066, 10.1016/j.cell.2020.02.041. 32164908 PMC9358771

[65] B. Shi , A. Qu , Z. Li , et al., “Chiral Intranasal Nanovaccines as Antivirals for Respiratory Syncytial Virus,” Advanced Materials 36, no. 41 (2024): 2408090, 10.1002/adma.202408090. 39221522

[66] E.‐S. E. Mehana , A. F. Khafaga , and S. S. El‐Blehi , “The Role of Matrix Metalloproteinases in Osteoarthritis Pathogenesis: An Updated Review,” Life Sciences 234 (2019): 116786, 10.1016/j.lfs.2019.116786. 31445934

[67] H. S. Hwang , J. Baek , S.‐Y. Lee , et al., “Association between Circulating ECM‐Associated Molecules and Cardiovascular Outcomes in Hemodialysis Patients: A Multicenter Prospective Cohort Study,” Biomarker Research 12, no. 1 (2024): 22, 10.1186/s40364-023-00553-x. 38331932 PMC10854113

[68] G. Jin , M. Zou , L. Li , et al., “Corneal Biomechanics and Their Association with Severity of Lens Dislocation in Marfan Syndrome,” International Ophthalmology 44, no. 1 (2024): 148, 10.1007/s10792-024-03079-9. 38502381

[69] D. Scheibenberger , A. Frings , J. Steinberg , et al., “Ocular Manifestation in Marfan Syndrome: Corneal Biomechanical Properties Relate to Increased Systemic Score Points,” Graefe's Archive for Clinical and Experimental Ophthalmology 256, no. 6 (2018): 1159–1163, 10.1007/s00417-018-3946-4. 29525839

[70] Q.‐Y. Huo , R.‐Z. Zhang , W.‐N. Jia , et al., “Corneal Biomechanics Are Associated with FBN1 Mutations in Patients with Marfan Syndrome and Ectopia Lentis,” Investigative Ophthalmology & Visual Science 66, no. 3 (2025): 23, 10.1167/iovs.66.3.23. PMC11905578 40062814

