# Supplementary material for: Chiral Nanoparticles Suppress Inflammatory Infiltration to Promote Extracellular Matrix Remodeling for Ectopia Lentis Therapy
Source: Adv Sci (Weinh). 2026 Jul 27;13(58):e22741. doi: 10.1002/advs.202522741 (PMC13403380; doi:10.1002/advs.202522741)
Supplement: Supplementary file 1 [file ADVS-13-e22741-s002.docx]

**Supporting Information**

**Chiral Nanoparticles Suppress Inflammatory Infiltration to Promote Extracellular Matrix Remodeling for Ectopia Lentis Therapy**

YinuoWen ^1,5,6,7#^, Yan Yang ^4,8#^, Haihan Gao ^2#^, Yan Liu ^1,5,6,7#^, Xinyue Wang ^1,5,6,7#^, Linghao Song ^1,5,6,7^, Ruohong Li ^1,5,6^, Ao Miao ^1,5,6^, Jie Xu ^1,5,6^, Zhennan Zhao ^1,5,6^, Dan Li ^1,5,6^, Shenjie Peng ^7^, Linzhao Li ^7^, Min Zhang ^1,5,6^, Yonghang Liu ^3^*, Liren Wang ^2,9^*, Tianhui Chen ^1,5,6^*, Yongxiang Jiang ^1,5,6^*

^1^ Eye Institute and Department of Ophthalmology, Eye & ENT Hospital, Fudan University, Shanghai, 200031, China

^2^ Department of Sports Medicine, Department of Orthopedic Surgery, Shanghai Institute of Microsurgery on Extremities, Shanghai Sixth People's Hospital Affiliated to Shanghai Jiao Tong University School of Medicine, Shanghai, 200233, China

^3^ School of Pharmacy and State Key Laboratory of Quality Research in Chinese Medicine, Macau University of Science and Technology, Macao, 999078, China

^4^ Guangdong-Hong Kong Joint Laboratory for Water Security, Beijing Normal University, Zhuhai, 519087, China

^5^ Key laboratory of Myopia and Related Eye Diseases, NHC; Key laboratory of Myopia and Related Eye Diseases, Chinese Academy of Medical Sciences, Shanghai, 200031, China

^6^ Shanghai Key Laboratory of Visual Impairment and Restoration, Shanghai, 200031, China

^7^ Shanghai Medical College (SHMC), Fudan University, Shanghai, China

^8^ Center for Water Research, Advanced Institute of Natural Sciences, Beijing Normal University, Zhuhai, 519087, China

^9^ National Center for Translational Medicine (Shanghai) SHU Branch, Shanghai University, Shanghai, 200444, China

*Corresponding authors.

E-mail: liuyh1996@163.com (Y.L.); wangliren13@163.com (L.W.); chentianhui97@163.com (T.C.); yongxiang_jiang@163.com (Y.J.).

^#^ These authors contributed equally.

**List of Supplementary figures**

Figure S1. Size variations of L-CN at different pH.

Figure S2. Cell viability (%) of HCECs and HTMCs incubated with L-CN, D-CN and DL-CN at different concentrations.

Figure S3. Biocompatibility of L-CN, D-CN and DL-CN.

Figure S4. Relative fluorescence intensity of IF of FBN1, iNOS and CD86.

Figure S5. Multi-omics analysis of selected capsular tissues between CC and EL patient samples.

Figure S6. *In vivo* metabolic process of L-CN, D-CN and DL-CN.

Figure S7. RT-qPCR analysis of the effects of L-CN, D-CN, and DL-CN on the expression of M_1_ polarization marker IL-1β.

Figure S8. Quantitative analysis of M_2_-associated surface marker expression by Western blot.

Figure S9. Concentrations of TGF-β, IL-10, and IL-13 in the supernatant from each group.

Figure S10. Validation of FBN1-targeting siRNA’s biological effects.

Figure S11. RNA-seq indicated that the NF-κB signaling pathway served as the specific molecular target of L-CN.

Figure S12. Histograms of diameter distribution from each group.

Figure S13. Behavioral tests.

Figure S14. Single-cell transcriptomic profiling of rat ciliary body-zonule complex tissues in model and L-CN groups.

Figure S15. Single-cell transcriptomic profiling of immune cells in model and L-CN groups.

Figure S16. Reshaping of macrophage subclusters in ciliary body-zonule complex tissues by L-CN injection.

Figure S17. *In vivo* biocompatibility after L-CN intravitreal injection.

**List of Supplementary tables**

Table S1. Fiber Maturing Score

Table S2. Sequences for qPCR

**Supplementary methods**

**Figure S1.** Size variations of L-CN at different pH.

**Figure S2.** Cell viability (%) of HCECs and HTMCs incubated with L-CN, D-CN and DL-CN at different concentrations. A, B) CCK-8 assays of HCECs and HTMCs at day 1 (n = 5). C, D) Calcein-AM/PI double staining of HCECs and HTMCs at day 3 (n = 3). Scale bar, 200 μm. All data were presented as mean ± SD. Statistical significance was determined using one-way analysis of variance (ANOVA) with Tukey’s multiple comparisons.

**Figure S3.** Biocompatibility of L-CN, D-CN and DL-CN. A) Live/Dead staining of HLECs in different groups at day 1, day 3, and day 5 (n = 5). Scale bar, 250 μm. B-D) Cell migration evaluation through scratch test (n = 5). Scale bar, 250 μm. E, F) Cell migration evaluation through transwell analysis (n = 5). Scale bar, 50 μm. Statistical analyses were performed using One-way ANOVA followed by Tukey’s multiple comparisons test. Data are presented as mean ± SD.

**Figure S4.** Relative fluorescence intensity of IF of FBN1 (A), iNOS (B), and CD86 (C) (n = 3). Statistical analyses were performed using unpaired two-tailed Student’s t test. Data are presented as mean ± SD.

**Figure S5.** Multi-omics analysis of selected capsular tissues between CC and EL patients. A) Principal component analysis (PCA) results for differentially expressed genes between CC and EL patient samples. B) Volcano plot illustrating DEGs in CC versus EL cohorts. C) Heat map highlighting genes differentially expressed (red for upregulated, blue for downregulated) between CC and EL patient samples. D) KEGG Sankey diagram corresponding to pathways enriched for genes differentially expressed between CC and EL patient samples. E) GO enrichment circus results for differentially expressed genes between CC and EL patient samples. F) PCA of differentially abundant proteins identified in CC and EL patients. G) Scatter plot of differentially abundant proteins identified in CC and EL patients. H) GO enrichment analysis of differentially abundant proteins identified in EL patients. I) Volcano plot comparing metabolite expression in CC versus EL patients. J) PCA results for differentially metabolite expression in CC versus EL patients. K) Orthogonal Partial Least Squares-Discriminant Analysis (OPLS-DA) results for differentially metabolite expression in CC versus EL patients.

**Figure S6.** *In vivo* metabolic process of L-CN, D-CN and DL-CN. A) *In vivo* real-time fluorescence imaging of rats treated with D-CN and DL-CN over a 17-day observation period. B) Quantitative analysis of intraocular fluorescence intensity of L-CN, D-CN, and DL-CN at indicated time points following intravitreal injection (n = 3). All data were presented as mean ± SD.

**Figure S7.** RT-qPCR analysis of the effects of L-CN, D-CN, and DL-CN on the expression of M_1_ polarization marker IL-1β (n = 5). Statistical analyses were performed using One-way ANOVA followed by Tukey’s multiple comparisons test. Data are presented as mean ± SD.

**Figure S8.** Quantitative analysis of M_2_-associated surface marker expression of Arg-1 (A), CD163 (B), and CD206 (C) by western blot (n = 3). Statistical analyses were performed using One-way ANOVA followed by Tukey’s multiple comparisons test. Data are presented as mean ± SD.

**Figure S9.** Concentrations of TGF-β (A), IL-10 (B), and IL-13 (C) in the supernatant from each group (n = 5). All data were presented as mean ± SD. Statistical significance was determined using one-way ANOVA with Tukey’s multiple comparisons.

**Figure S10.** Validation of *FBN1*-targeting siRNA’s biological effects. A) Relative fluorescence intensity of FBN1 in HLECs after siRNA-mediated knockdown (n = 3). B, C) Western blot of FBN1 expression in HLECs under different induction conditions (n = 3). RNA Sequencing of si*FBN1* and si*NC*. D) PCA results for two groups. E) Heatmap of differentiated gene expression. F) Volcano plot of differentiated gene expression. G) GO enrichment analysis. H) Relative fluorescence intensity of iNOS in RAW 264.7 cells in different groups (n = 3). I) Relative fluorescence intensity of FBN1 in HLECs in different groups (n = 3). Statistical analyses were performed using One-way ANOVA followed by Tukey’s multiple comparisons test (A) and unpaired two-tailed Student's t test (C, H, I). Data are presented as mean ± SD.

**Figure S11.** RNA-seq indicated that the NF-κB signaling pathway served as the specific molecular target of L-CN. A) PCA results for three groups. B) Heatmap of differential gene expression for two groups. C) Volcano plot of differential gene expression. D) GO enrichment analysis. E) Top 30 enriched genes identified by GSEA in GO enrichment analysis.

**Figure S12.** Histograms of diameter distribution from each group (n = 5).

**Figure S13.** Behavioral tests. A) Total time in all quadrants in MWM (n = 5). B, C) ERG changes before treatment (n = 3). Statistical analyses were performed using One-way ANOVA followed by Tukey’s multiple comparisons test (A) and two‐way ANOVA with Dunnett’s multiple comparisons test (B, C). Data are presented as mean ± SD. ns, not significant.

**Figure S14.** Single-cell transcriptomic profiling of rat ciliary body-zonule complex tissues in EL and L-CN groups. A) UMAP visualization of all cells in EL and L-CN groups, colored according to cell clusters. B) Dot plot for expression of marker genes in each cell cluster. C-F) Volcano maps showing the DEGs in different cell clusters. G) Violin plots of specific gene expression between EL and L-CN groups.

**Figure S15.** Single-cell transcriptomic profiling of immune cells in EL and L-CN groups. A) UMAP visualization of immune cells of EL and L-CN groups, colored according to cell clusters. B) Dot plot for expression of marker genes in each cell cluster. C-E) Volcano maps showing the DEGs in different cell clusters. F, G) GO enrichment analysis of up-regulated genes in EL and L-CN groups. H) Violin plots of specific gene expression in M_0_, M_1_ and M_2_ macrophages. I) Bar plot showing the comparison of the macrophage subtypes between two groups.

**Figure S16.** Reshaping of macrophage subclusters in ciliary body-zonule complex tissues by L-CN injection. A) The developmental trajectory of macrophages from all samples colored according to pseudotime. B) Ridge plots showed the macrophages’ development from all samples. C) Pseudo-heatmap showed the significant genes correlated with the development of macrophages in fate 1 and fate 2.

**Figure S17.** *In vivo* biocompatibility after L-CN intravitreal injection. A) OCT evaluation of retinal structure in rats injected with L-CN for 8 weeks (n = 3). B) H&E staining of the heart, liver, spleen, lung, and kidney in rats 8 weeks after L-CN injection (n = 3). Scale bar, 50 μm. C-G) Peripheral blood biochemical analysis and liver/kidney function evaluation in rats following 8 weeks of L-CN injection (n = 3).

| **Table S1.** Fiber Maturing Score | | | |  |
| --- | --- | --- | --- | --- |
|  | 1 | 2 | 3 | 4 |
| Cellularity | Marked | Moderate | Mild | Minimal |
| Proportion of fibers of large diameter characteristic of mature zonular fibers | <25% | 25-50% | 50-75% | >75% |
| Proportion of parallel fibers | <25% | 25-50% | 50-75% | >75% |
| Vascularity | Marked | Moderate | Mild | Minimal |

| **Table S2.** Sequences for qPCR | | | |
| --- | --- | --- | --- |
| Species | Gene name | Forward primer | Reverse primer |
| human | *MFAP2* | CACGTCCAGTACACCCACTAT | CGAGGAGTCACCTCTTGATAA |
| human | *FBN1* | GCTTCAAATGTCTGTGTCCAG | TCCTTCAAACTTCGCATAACA |
| human | *CD86* | CCTCTGTCAGGGTCAGTAAGG | TCCAGGTTCTATCTCTGCCTC |
| human | *iNOS* | GTGCTCTTTGCCTGTATGCTG | TGATTTTCCTGTCTCTGTCGC |
| human | *CD206* | GACCTCACAAGTATCCACACC | CCCATCACTCCACTCAAAGTA |
| human | *TNF-α* | CTGTGAGGAGGACGAACATCCA | GGGTGTCTGAAGGAGGGGGTAAT |
| human | *Arg-1* | CTAGGAATTGGCAAGGTGATG | GTGTGAAAGATGGGTCCAGTC |
| human | *MMP7* | ACCGCATATTACAGTGGATCG | TTCCCCATACAACTTTCCTGA |
| human | *MMP9* | GCTGGGCTTAGATCATTCCTC | ATTCACGTCGTCCTTATGCAA |
| human | *MMP13* | GCAGTCTTTCTTCGGCTTAGA | TTGTATTCACCCACATCAGGA |
| human | *GAPDH* | CCTCTGACTTCAACAGCGACA | ATGAGCTTGACAAAGTGGTCGT |
| rat | *FBN1* | GGGAGTGTACCAACACGGTC | CCATCGGGAGAGGTGTAAAAG |
| rat | *MFAP2* | GTGAAGAACAGTACCCATGTACC | GTAGAAGCAGACCTCATTGAGAC |
| rat | *iNOS* | CTCAGGCTTGGGTCTTGTTAG | TCTGTGACTTTGTGCTTCTGC |
| rat | *CD86* | CAACTAATGAGTATGGCGACA | GGGAATGGAAGAGATAGGCT |
| rat | *CD206* | CCTTCTGTGCCTATCTCTCCA | TATTTCTCTGCTTCGTGCCAT |
| rat | *Arg-1* | CGGGAAGGTAATCATAAGCCA | GTTCTGTTCGGTTTGCTGTGA |
| rat | *TNF-α* | CCCCAATCTGTGTCCTTCTAACT | CGTGTGTTTCTGAGCATCGTA |
| rat | *IL-6* | AATCTGCTCTGGTCTTCTGGA | ATTGCTCTGAATGACTCTGGC |
| rat | *IL-10* | TGCAGGACATAAATAGAGCTTC | GGAGAGAGGTACAAACGAGG |
| rat | *MMP7* | TCACCTACAGAATCGTGTCCT | CCCAACTAACCCTCTTGAAGT |
| rat | *GAPDH* | ACGGGAAACCCATCACCATC | CACGACATACTCAGCACCAGCA |
| mouse | *iNOS* | CAACAGGAACCTACCAGCTCACT | AGCCTGAAGTCATGTTTGCCG |
| mouse | *TNF-α* | CCCTCACACTCACAAACCACC | CTTTGAGATCCATGCCGTTG |
| mouse | *IL-6* | AGCCCACCAAGAACGATAG | GGTTGTCACCAGCATCAGT |
| mouse | *CD86* | TCTCCAACAGCCTCTCTCTTT | ATCTTCATTGACTCCGTTTCC |
| mouse | *CD206* | AGGGAAGAGAAGAAGATCCAG | TGGGAGAAGATGAAGTCAAAC |
| mouse | *Arg-1* | GCCAGGGACTGACTACCTTAA | AGTTCTGTCTGCTTTGCTGTG |
| mouse | *IL-10* | GGAAAACCTCGTTTGTACCT | GGGCTTCTTTCTAAATAGTTCAC |
| mouse | *GAPDH* | CCTCGTCCCGTAGACAAAATG | TGAGGTCAATGAAGGGGTCGT |

**Supplementary methods**

1. *RNA-Seq analysis*

For RNA-seq, RNA was extracted using a high-stringency protocol with guanidine isothiocyanate lysis buffer, proteinase K digestion, and DNase treatment, followed by silica-membrane purification. The process was automated using a nucleic acid extraction system, ensuring effective protein degradation and genomic DNA removal. Purified RNA was quantified using a Nanodrop 8000 spectrophotometer (Thermo Fisher, USA) and assessed for quality with an Agilent 4200 Bioanalyzer (Agilent, USA). Poly(A) mRNA was enriched using oligo(dT) magnetic beads, fragmented (~300 nt) with divalent cation buffer, and reverse transcribed using random hexamers (SuperScript IV, Thermo Fisher). The second-strand cDNA was synthesized, followed by end repair, adapter ligation (TruSeq, Illumina), and PCR amplification (KAPA HiFi, Roche). Paired-end sequencing was performed on an Illumina NovaSeq 6000 platform (Majorbio, Shanghai). Gene expression analysis, including PCA, heatmaps, volcano plots, GO enrichment, and KEGG pathway analysis, was conducted using R (version 2024.4.4.1).

2. *Aqueous Humor Protein Microarray Analysis*

Protein expression profiling of aqueous humor was performed using the Quantibody® Human Cytokine Array 2000 (QAH-CAA-2000, RayBiotech, Peachtree Corners, GA, USA). All procedures were carried out according to the manufacturer’s instructions. Briefly, glass array slides were equilibrated to room temperature and blocked with Sample Diluent for 1 h. Undiluted aqueous humor samples (100 μL per well) and serially diluted cytokine standards were then added to the array chambers and incubated overnight at 4 °C. After washing with Wash Buffer I and Wash Buffer II, the arrays were incubated with a cocktail of biotinylated detection antibodies for 2 h at room temperature, followed by incubation with Cy3-conjugated streptavidin for 1 h in the dark. Between each incubation step, the slides were washed according to the manufacturer’s protocol. Fluorescence signals were acquired using an InnoScan 300 Microarray Scanner (Innopsys, Carbonne, France) with a 532-nm excitation laser at a resolution of 10 μm. Raw fluorescence intensities were background-corrected and normalized using RayBiotech Quantibody® analysis software. Protein concentrations were calculated from standard curves generated using serially diluted recombinant protein standards. PCA, hierarchical clustering, volcano plots, GO enrichment analysis, and KEGG pathway enrichment analysis were subsequently performed using R (version 2024.4.4.1).

3. *Non-Targeted Metabolomics*

A 30 µL sample was combined with an equal volume of ice-cold acetonitrile (1:1, v/v), vortexed for 1 min, and centrifuged at 15,000× g for 10 min (4℃). The supernatant (50 µL) was transferred to conical-bottom autosampler plates, with 20 µL injected for analysis. Metabolite separation was performed using a Thermo Ultimate Dionex 3000 ultra-high-performance liquid chromatography (UHPLC) system (Thermo Fisher Scientific, USA), equipped with dual separation modes. Polar metabolites were separated on a ZIC-HILIC column (50 × 4.6 mm, 3.5 µm, Merck, Germany) with a binary gradient of 0.1% formic acid in ultrapure water and acetonitrile. Non-polar metabolites were separated using a Purospher Star C18 column (50 × 4.6 mm, 3 µm, Merck, Germany). The mobile phase flow rate was 500 µL/min with strict temperature control, and 20 µL was injected for untargeted metabolomics. Eluates were analyzed using a high-resolution Orbitrap Fusion™ Tribrid™ Mass Spectrometer (Thermo Fisher Scientific, USA) with electrospray ionization in both polarities. Full-scan MS spectra (m/z 70 - 700) were acquired at 120,000 resolution (at m/z 200) (AGC 4×10⁵, 50 ms max injection) with dynamic exclusion (8 s, ± 10 ppm). Data-dependent MS² scans were performed using high collision dissociation (HCD) at 15,000 resolution (at m/z 200). Acquisitions were controlled by Xcalibur™ (v4.3, Thermo Fisher Scientific, USA), with 0.6 s cycle times. Data were normalized using variance-stabilizing transformation and Pareto scaling. Statistical analysis included univariate analysis (Student’s t-test with false discovery rate (FDR) correction, *q* < 0.05) and multivariate pattern recognition (orthogonal partial least squares-discriminant analysis, OPLS-DA) using MetaboAnalyst 5.0 (Xia Lab, McGill University). PCA and OPLS-DA were used to identify discriminative features, which were matched with the NIST library and validated through relevant references.

4. *Synthesis of PAU-g-L-Cys*

Polycaprolactone diol (PCL-OH, 1 eq) and hexamethylene diisocyanate (HDI, 2 eq) were precisely weighed and transferred into a three-necked flask under a nitrogen atmosphere. The mixture underwent prepolymerization at 80 ℃ for 2 hours. Subsequently, dimethylolbutanoic acid (DMBA) dissolved in dimethyl sulfoxide (DMSO) was added, and the reaction continued for 3 h to achieve chain extension. At room temperature, N-ethyl-N’-(3-dimethylaminopropyl) carbodiimide hydrochloride (EDC, 1.2 eq) and N-hydroxysulfosuccinimide (NHS) were sequentially introduced to activate the carboxyl groups. L-cysteine (L-Cys, 2 eq) was then added, and the reaction was allowed to proceed for 24 h. The product was purified by washing with deionized water for 3 days and lyophilized to obtain the L-configuration PAU-g-L-Cys elastomer, with a yield of 92.6%.

5. *Preparation of L-CN*

To prepare the L-CN nanoparticles, 25 mg of PAU-g-L-Cys elastomer was dissolved in 1.5 mL dichloromethane and slowly added dropwise to a 1% polyvinyl alcohol (PVA) aqueous solution. The mixture was subjected to probe sonication (100 kW, 160 s) for dispersion, followed by evaporation of the dichloromethane under stirring for 6 h. Upon complete solvent removal, the nanoparticles were collected by centrifugation at 4 ℃ and washed three times with phosphate-buffered saline (PBS) to yield the final L-CN product.

6. *Primary Culture of BMDMs*

Femurs and tibiae were aseptically dissected, and bone marrow was flushed with PBS supplemented with 1% penicillin-streptomycin. Erythrocytes were lysed using ACK lysis buffer (Yeasen, China) for 5 minutes at room temperature. The resulting cells were cultured in high-glucose Dulbecco’s modified Eagle’s medium (H-DMEM; Gibco, USA) supplemented with 10% fetal bovine serum (FBS) and 40 ng/mL recombinant mouse macrophage colony-stimulating factor (M-CSF; Novoprotein, China) at 37 ℃ in a 5% CO₂ incubator for 7 days, with medium refreshed on day 4. Adherent cells exhibiting typical macrophage morphology were then harvested for subsequent experiments.

7. *Scratch Wound Healing Assay*

1 × 10⁶ HLECs were plated into six-well tissue culture plates with 2 mL of complete medium and cultured overnight to near confluence. Prior to the scratch, cells were serum-starved for 2 h. A sterile P-200 pipette tip was used to create an artificial wound by scratching across the center of each well. The plates were then incubated in a CO₂ incubator and imaged after 12 h and 24 h post-scratch. Cell migration was observed and recorded using a fluorescence microscope (Olympus, Tokyo, Japan) at each time point. The migration rate was calculated as the ratio of the migrated area to the original area, analyzed using ImageJ software.

8. *Transwell Migration Assay*

HLECs were seeded in the upper chamber of transwell inserts (Corning, USA) with serum-free α-Minimum Essential Medium (α-MEM). Complete α-MEM in the lower chamber was considered the control group, while the experimental group included cells cultured in complete medium with 200 µg/mL D-CN, L-CN and DL-CN. After 24 h of incubation, the inserts were fixed with 4% paraformaldehyde (PFA) for 30 minutes, stained with crystal violet (Beyotime, China) for 15 minutes, and photographed under a light microscope.

9. *Cell Viability and Toxicity Assays*

HLECs (1 × 10⁵ cells/mL) were seeded into 96-well plates (for CCK-8 assays) and 24-well plates (for Live/Dead assays), and incubated with varying concentrations of D-CN, L-CN, and DL-CN (50 µg/mL, 100 µg/mL, 200 µg/mL, and 500 µg/mL) for 1, 3, and 5 days. The control group was cultured in complete α-MEM. For the CCK-8 assay, cells were incubated with 10% CCK-8 solution in complete medium for 1 h at 37 ℃ in 5% CO₂, followed by absorbance measurement at 450 nm using a microplate reader (MPR-A9600, Thomas Scientific, USA). For the Live/Dead assay, cells were stained with calcein-AM and propidium iodide (PI) as the manufacturer’s instructions. Working solutions were prepared by diluting stock solutions 1:1000 in buffer. After a 30-minute incubation (37 ℃, dark conditions), cells were imaged using a DMi8 microscope (Leica, Germany). Viable and dead cells were quantified using ImageJ software (National Institutes of Health, USA).

10. *Phagocytosis Assays*

RAW 264.7 cells were seeded into 24-well plates (for IF staining) and 6-well plates (for flow cytometry). After the medium was aspirated, cells were incubated with 200 µg/mL chiral nanoparticles in serum-free DMEM for 2 h or 12 h at 37 ℃ in 5% CO₂. Uptake was terminated by washing with PBS before downstream analysis. Following fixation, cells were stained with phalloidin and imaged using a DMi8 microscope (Leica, Germany). ImageJ software (National Institutes of Health, USA) was used for quantification. For flow cytometry, the digested cell suspension was immediately processed and analyzed with FlowJo software (v10.8.1, USA).

For TEM, cell suspensions were fixed in freshly prepared 2% (w/v) glutaraldehyde at 4℃ for 24 h for optimal ultrastructural preservation. After rinsing with cold PBS (3 × 15 min), secondary fixation was performed using 1% (w/v) osmium tetroxide at 4℃ for 90 minutes. Samples were infiltrated with a mixture of Epon-Araldite resin and propylene oxide (1:2, 1:1, and 2:1 ratios), each for 24 h. The specimens were then embedded in pure Epon-Araldite resin at 60℃ for 48 h. Thin sections (~ 0.1 µm) were cut and examined under a Talos L120C G2 transmission electron microscope (Thermo Fisher, USA).

11. *In Vivo Biodistribution Evaluation*

Nine rats of the EL model were randomized into three treatment groups and administered 8 µL of Cy7-labeled L-CN, D-CN or DL-CN via intravitreal injection. Postinjection, the *in vivo* fluorescence imaging system (IVScope8200, Clinx, China) was used to capture and record the fluorescence signals of rats’ eyes in each group at designated time intervals up to 17 days after administration.

12. *Macrophage Polarization Assays*

M_1_ Polarization: RAW 264.7 cells were seeded at a density of 1 × 10⁵ cells per well in a 6-well plate. The negative control group was cultured in complete DMEM, while the experimental groups were incubated in complete DMEM with 200 ng/mL LPS (Sigma, USA) and treated with D-CN, L-CN, or DL-CN.

M_2_ Polarization: RAW 264.7 cells were seeded at a density of 1 × 10⁵ cells per well in a 6-well plate. The positive control group was cultured in complete DMEM supplemented with 40 ng/mL IL-4 and IL-13 (Yeasen, China). The experimental groups were cultured in complete DMEM containing 40 ng/mL IL-4 and IL-13, along with D-CN, L-CN, or DL-CN.

13. *siRNA and Transient Transfection*

The sequences for HLECs siRNA1 were listed as follows: 5’ GAAAGACACUCCAACUAAUTT‐3’ (sense), 5’‐ AUUAGUUGGAGUGUCUUUCTT‐3’ (antisense); the sequences for HLECs siRNA2 were 5’‐ CACAUUACCAGGAGCACAATT‐3’(sense), 5’‐ UUGUGCUCCUGGUAAUGUGTT‐3’ (antisense); the sequences for HLECs siRNA3 were 5’‐ GCUCAAUGCUUCGAAUUCATT‐3’ (sense), 5’‐ UGAAUUCGAAGCAUUGAGCTT‐3’ (antisense).

14. *Real-time quantitative polymerase chain reaction (RT-qPCR)*

Total RNA was extracted from cultured cells using the EZ-press RNA Purification Kit (EZBioscience, USA) according to the manufacturer’s instructions. First-strand cDNA synthesis was performed using 1 µg of total RNA and a reverse transcription kit (EZBioscience, USA). Quantitative PCR (qPCR) was carried out on a LightCycler 480 system (Roche, Germany) with SYBR Green qPCR Master Mix (EZBioscience, USA), under the following conditions: 95 ℃ for 5 minutes, followed by 40 cycles of 95 ℃ for 15 seconds and 60 ℃ for 30 seconds. Glyceraldehyde-3-phosphate dehydrogenase (GAPDH) served as the reference gene. Gene-specific primers (Sangon Biotech, China) were used to amplify the following markers: *GAPDH*, Tumor Necrosis Factor-alpha (*TNF-α*), inducible nitric oxide synthase (*iNOS*), *CD86*, Interleukin (*IL*)*-6*, matrix metalloproteinases (*MMP*)*-7*, *-9*, and *-13,* arginase-1 (*Arg-1*), *CD206*, *IL-10*, fibrillin-1 (*FBN1*), and Microfibril-Associated Protein 2 (*MFAP2*). Primer sequences are provided in Table S2. Relative gene expression was calculated using the 2^(-ΔΔCt) method, with three technical replicates for each sample.

*15. Flow cytometry*

HLECs and RAW 264.7 cells, and BMDMs were seeded at a density of 1 × 10⁶ cells per well in six-well plates and incubated overnight. The cells were then treated with 200 µg/mL of D-CN, L-CN, or DL-CN for 24 h. After treatment, cells were harvested and processed into a single-cell suspension. The cells were then stained with Annexin V/PI (Elabscience, China, E-CK-A219), PE-conjugated anti-iNOS antibody (BioLegend, USA, 696806, 1:100), and APC-conjugated anti-CD206 antibody (BioLegend, USA, 141708, 1:200), following the manufacturer’s instructions. After staining, samples were washed twice with PBS and analyzed by fluorescence-activated cell sorting (FACS). Flow cytometry was performed on a Canto II flow cytometer (BD, USA) equipped with 488 nm and 633 nm lasers. Compensation was performed using single-stained controls, and a minimum of 10,000 events were acquired per sample. Data were processed using FlowJo software (v10.8.1, USA).

*16. Immunofluorescence staining (IF)*

After aspiration of the culture medium, cells were washed three times with PBS and fixed with freshly prepared 4% PFA in PBS for 30 minutes at room temperature. Following fixation, cells were permeabilized with 0.5% Triton X-100 in PBS for 15 minutes and subsequently blocked with 5% bovine serum albumin (BSA) in PBS for 30 minutes at room temperature. Primary antibodies, diluted in blocking buffer, were incubated overnight at 4 ℃: iNOS (Thermo Fisher, cat# MA5-17139), iNOS (Servicebio, cat# GB11119), CD86 (Novusbio, cat# IMG-6882A), ARG1 (Servicebio, cat# GB11285), CD206 (Cell Signaling Technology, cat# 24595), FBN1 (Thermo Fisher, cat# PA5-99225), MFAP2 (Abcam, cat# ab203828), and NF-κB p65 (Beyotime, cat# AB2020). After primary antibody incubation, cells were washed three times with PBS and incubated with Alexa Fluor® 594-conjugated Goat Anti-Mouse IgG (H+L) secondary antibody (Cell Signaling Technology, cat# 8890), Alexa Fluor® 488-conjugated Goat Anti-Mouse IgG (H+L) secondary antibody (Cell Signaling Technology, cat# 4408), Alexa Fluor® 594-conjugated Goat Anti-Rabbit IgG (H+L) secondary antibody (Cell Signaling Technology, cat# 8889), Alexa Fluor® 488-conjugated Goat Anti-Rabbit IgG (H+L) secondary antibody (Cell Signaling Technology, cat# 4412) for 2 h at room temperature protected from light. For cytoskeletal visualization, cells were co-stained with phalloidin-iFluor 488 reagent (Abcam, cat# ab176753) and phalloidin-iFluor 594 reagent (Abcam, cat# ab176757) for 30 min, and nuclei were counterstained with 4’,6-diamidino-2-phenylindole (DAPI) hydrochloride for 10 minutes, followed by visualization using a DMi8 microscope (Leica, Germany) and confocal fluorescence microscope (Leica SP8, Germany).

*17. Western blot*

Cells were lysed in RIPA buffer (Beyotime, China) supplemented with a protease inhibitor cocktail (Beyotime, China) for 30 minutes on ice. The lysates were then centrifuged at 12,000× g for 15 minutes at 4 ℃, and the supernatants were collected for protein quantification using the BCA Protein Assay Kit (Beyotime, China), following the manufacturer’s instructions. Equal amounts of protein from each sample were separated by SDS-PAGE and Phos-tag™ SDS-PAGE, transferred onto PVDF membranes. The membranes were incubated overnight at 4 ℃ with primary antibodies diluted in blocking buffer, with gentle agitation: iNOS (Servicebio, cat# GB11119), CD86 (Novusbio, cat# IMG-6882A), IL-1β (Proteintech, cat# 26048-1-AP), ARG1 (Servicebio, cat# GB11285), CD206/MRC-1 (Cell Signaling Technology, cat# 24595S), CD163 (MedChemExpress, cat# HY-P81177), FBN1 (Thermo Fisher, cat# PA5-99225), NF-κB p65 (Cell Signaling Technology, cat# 8242), Phospho-NF-κB p65 (Cell Signaling Technology, cat# 3033), Phospho-IκBα (Cell Signaling Technology, cat# 2859), Lamin B (Cell Signaling Technology, cat# 13435), GAPDH (Cell Signaling Technology, cat# 2118), and β-actin (Cell Signaling Technology, cat# 4970). After washing with TBST (3 × 10 min), membranes were incubated with Peroxidase AffiniPure® Goat Anti-Mouse IgG (H+L) (Jackson, cat# 115-035-003) and Peroxidase AffiniPure® Goat Anti-Rabbit IgG (H+L) (Jackson, cat# 111-035-003) for 1 h at room temperature. Signals were captured using a gel imaging system (Thermo Fisher, USA).

*18. Enzyme-linked immunosorbent assay (ELISA)*

The supernatants from M_1_ and M_2_ macrophages in different experimental groups were collected. The concentrations of TNF-α (A1010A0203), IL-1β (A1010A0201), CCL-2 (A1010A0245), IL-6 (A1010A0202), TGF-β (MEC1012), IL-10 (EK210) and IL-13 (EK213) in the supernatants were measured using ELISA kits (BioTNT, China), following the manufacturer’s instructions.

19. *Animal Preparation and Surgical Procedure*

Male Sprague-Dawley rats (8 weeks old) were purchased from JieSiJie Laboratory Animal Co., Ltd. (Shanghai, China). Anesthesia was induced via intraperitoneal injection of 10% pentobarbital sodium. Rats were randomly allocated to each experimental group to minimize allocation bias (50 per group). Prior to surgery, the rats were treated with 0.25% tropicamide eye drops at 10-minute intervals (three applications in total) to induce pupil dilation, followed by proparacaine hydrochloride eye drops with each dilation. A 2 mg/mL α-chymotrypsin solution was prepared by dissolving an appropriate amount of chymotrypsin powder in 1 mM HCl and heavy sucrose medium, then stored at 4℃ for subsequent use. To prevent dilution of the α-chymotrypsin solution, excess eye drops were carefully removed from the corneal surface and conjunctival sac using sterile cotton swabs. For posterior zonule fiber lysis, 6 µL of the α-chymotrypsin solution was injected through a ~1 mm incision at the 9 o’clock position of the corneoscleral limbus. The solution was delivered through the sclera and choroid into the midregion of the pars plana, with the ocular globe positioned to allow gravitational deposition directly onto the zonular fibers. Model success was confirmed when animals exhibited the following features: (i) reproducible and stable phenotypic characteristics consistent with lens dislocation; and (ii) absence of spontaneous recovery during the early post-induction observation period. Only animals meeting all predefined criteria were included in subsequent analyses.

20. *Tissue Collection and Dissection*

At 21 days post-treatment, rats were euthanized with an overdose of 10% pentobarbital sodium. Following enucleation, the cornea and iris tissues were carefully excised to expose the anterior lens capsule. A circumferential incision was made 2.0 mm posterior to the limbus to reveal the posterior surface of the lens. Residual vitreous was meticulously removed, and the intact lens-zonular fiber-scleral ring complex was subsequently harvested for further analysis.

21. *Histological Analysis*

The lens-zonular fiber-scleral ring complex was fixed in 4% PFA for 24 h, followed by decalcification in 10% EDTA for 14 days. After dehydration, the tissue was embedded in paraffin. H&E and Masson’s trichrome staining were performed according to standard protocols to assess the general histological structure of the zonular fibers. Fiber maturation was evaluated by two blinded observers using a modified scoring system, as previously described. Higher scores indicated improved zonular fiber construction.

22. *Single-cell RNA sequencing*

Cell viability typically exceeded 50%, ensuring reliable quality control and accurate enumeration of the single-cell suspension. Following quality control, single cells were randomly distributed into microwells by gravitational settling. Beads embedded with oligonucleotide barcodes were then introduced, allowing them to pair with individual cells. After cell lysis, the barcoded beads, which contained the captured mRNA, were retrieved, washed, and subjected to reverse transcription and Exonuclease I digestion. Single-cell transcriptomic sequencing libraries were generated following a series of PCR steps, including random priming and extension (RPE), RPE PCR, and whole-transcriptome amplification (WTA) index PCR, according to the manufacturer’s protocol. The normalized libraries were then sequenced on the NovaSeq Illumina platform (China) using a 150 bp paired-end configuration.

23. *Retinal Function Assessment*

Healthy male rats were selected for the study and anesthetized using both systemic and topical methods. An 8 µL intravitreal injection of L-CN was administered to the right eye in the temporal quadrant, while the left eye received an equal volume of PBS as a control. Retinal structures, along with visual function were evaluated 8 weeks post-injection using an OCT (ISOCT-II, Optoprobe).

*24.* *Behavioral Tests*

*24.1. Morris water maze (MWM)*

To assess visual function, rats were tested in the Morris water maze (MWM) apparatus using a visible escape platform (12 cm in diameter) positioned approximately 1 cm above the water surface. The circular pool was 160 cm in diameter, and the water temperature was maintained at 22 - 23 °C. The pool was divided into four quadrants (NE, NW, SE, and SW), and the platform was placed in the NE quadrant. Animal trajectories were recorded using the Tracking Master system (China). Each rat was gently placed into the water from a designated starting position and allowed up to 300 s to locate and climb onto the visible platform. The latency to reach the platform, swimming distance, and swimming speed were recorded as indicators of visual and motor performance. Rats that successfully climbed onto the platform were removed immediately and returned to their home cage. The visual environment remained constant throughout the experiment and consisted of geometric figures placed around the maze, surrounding walls of different colors, and the presence of experimenters. No pre-training or acquisition trials were conducted before testing.

*24.2. Optomotor Response (OMR)*

Visual acuity and contrast sensitivity were assessed using an OMR paradigm. The testing system consisted of four liquid-crystal display monitors arranged in a quadrangle, with a camera mounted above for video recording. Visual stimuli (drifting black-and-white gratings, 12° s⁻¹) were generated using the Psychopy toolbox. Gratings drifted alternately in clockwise or counterclockwise-direction, each presented for 10 s and repeated three times. Rats were habituated to remain on a pedestal (12 cm in height, 4 cm in radius) for 5 min on the day prior to testing. During experiments, rats were placed on the pedestal and allowed to move freely. A grey background was shown at the beginning of each session and replaced by drifting gratings once the rat became stationary. Trials were paused and resumed if the rat slipped or jumped off the pedestal. A valid OMR trial was defined as an angular head movement concordant with the direction of grating motion. An OMR was considered positive when at least one valid head-tracking trial was observed in both directions. For visual acuity testing, gratings were initially presented at 100% contrast and 0.1 cycles per degree. Spatial frequency was subsequently increased in 0.05 cycles per degree steps until no OMR was detected. The highest spatial frequency that elicited an OMR at 100% contrast was defined as the visual acuity. For contrast sensitivity testing, gratings were presented at eight spatial frequencies (0.031, 0.045, 0.064, 0.092, 0.130, 0.192, 0.272, and 0.350 cycles per degree), starting at 100% contrast. Following a positive OMR, contrast was decreased stepwise until no response was observed. The lowest contrast eliciting an OMR defined the contrast threshold for each spatial frequency, and contrast sensitivity was calculated as 1/threshold. Rats with glass implants that exhibited no OMR were assigned a contrast sensitivity value of zero.

*24.3. ERG testing*

The ERG signal acquisition followed the standardized protocol of the International Society for Clinical Electrophysiology of Vision (ISCEV). Following overnight dark adaptation, rats were anesthetized, a subcutaneous ground electrode was placed in the tail, and two reference electrodes were positioned beneath the bilateral cheek skin. Two gold wire loop electrodes were carefully placed on the corneal surface. Full-field ERG recordings were performed and the results from dark-adapted 1.0 ERG conditions were included in the subsequent analysis (n ≥ 3 per group). Both a-wave and b-wave amplitudes were systematically recorded and averaged for analysis.
